# Extracorporeal membrane oxygenation for cardiogenic shock: a meta-analysis of mortality and complications

**DOI:** 10.1186/s13613-022-01067-9

**Published:** 2022-10-05

**Authors:** Sasa Rajsic, Benedikt Treml, Dragana Jadzic, Robert Breitkopf, Christoph Oberleitner, Marina Popovic Krneta, Zoran Bukumiric

**Affiliations:** 1 Department of Anesthesiology and Intensive Care Medicine, Medical University Innsbruck, 6020 Innsbruck, Austria; 2 Anesthesia and Intensive Care Department, Pain Therapy Service, Cagliari University, Cagliari, Italy; 3 Institute for Oncology and Radiology of Serbia, Belgrade, Serbia; 4 Institute of Medical Statistics and Informatics, Faculty of Medicine, University of Belgrade, 11000 Belgrade, Serbia

**Keywords:** Adverse events, Cardiogenic shock, Complications, Extracorporeal life support, ECMO, Mortality, Venoarterial extracorporeal membrane oxygenation

## Abstract

**Background:**

Venoarterial extracorporeal membrane oxygenation (va-ECMO) is an advanced life support for critically ill patients with refractory cardiogenic shock. This temporary support bridges time for recovery, permanent assist, or transplantation in patients with high risk of mortality. However, the benefit of this modality is still subject of discussion and despite the continuous development of critical care medicine, severe cardiogenic shock remains associated with high mortality. Therefore, this work aims to analyze the current literature regarding in-hospital mortality and complication rates of va-ECMO in patients with cardiogenic shock.

**Methods:**

We conducted a systematic review and meta-analysis of the most recent literature to analyze the outcomes of va-ECMO support. Using the PRISMA guidelines, Medline (PubMed) and Scopus (Elsevier) databases were systematically searched up to May 2022. Meta-analytic pooled estimation of publications variables was performed using a weighted random effects model for study size.

**Results:**

Thirty-two studies comprising 12756 patients were included in the final analysis. Between 1994 and 2019, 62% (pooled estimate, 8493/12756) of patients died in the hospital. More than one-third of patients died during ECMO support. The most frequent complications were renal failure (51%, 693/1351) with the need for renal replacement therapy (44%, 4879/11186) and bleeding (49%, 1971/4523), bearing the potential for permanent injury or death. Univariate meta-regression analyses identified age over 60 years, shorter ECMO duration and presence of infection as variables associated with in-hospital mortality, while the studies reporting a higher incidence of cannulation site bleeding were unexpectedly associated with a reduced in-hospital mortality.

**Conclusions:**

Extracorporeal membrane oxygenation is an invasive life support with a high risk of complications. We identified a pooled in-hospital mortality of 62% with patient age, infection and ECMO support duration being associated with a higher mortality. Protocols and techniques must be developed to reduce the rate of adverse events. Finally, randomized trials are necessary to demonstrate the effectiveness of va-ECMO in cardiogenic shock.

**Supplementary Information:**

The online version contains supplementary material available at 10.1186/s13613-022-01067-9.

## Background

Extracorporeal membrane oxygenation (ECMO) is an advanced life support modality for critically ill patients with refractory respiratory or cardiac failure. The first reports of prolonged extracorporeal oxygenation of a patient with severe respiratory failure date from 1971 and present the beginning of ECMO support as we know it today [1, 2]. This temporary support for cardiorespiratory failure bridges time for recovery, permanent assistance, or transplantation. It is used as a last resort in severe respiratory failure as a venovenous (vv-ECMO) and in a cardiogenic shock as a venoarterial (va-ECMO) configuration [3]. In the last decades, ECMO support has been used increasingly in a variety of clinical presentations, like bridging to lung or heart transplant, resuscitation of patients with severe traumas, or extracorporeal-assisted rewarming (ECAR) of accidental hypothermia. The Extracorporeal Life Support Organization (ELSO) recommends the initiation of ECMO support in case of cardiorespiratory failure with a high risk of mortality (80%)[4].

Based on the data from 543 ELSO centers, more than 170,000 ECMOs were employed until the end of 2021. The number of ECMO support cases increased gradually in the last 10 years, especially since the outbreak of coronavirus disease 2019 (COVID-19). The reported survival rate to discharge or transfer of all adult ECMO patients was 49%, with 58% in case of respiratory failure and 45% for cardiac failure [5]. However, these data originate only from the ELSO registered centers, missing the data from other centers and introducing a potential selection bias.

The overall benefit, adverse events, and mortality rate are still the subject of discussion. Despite the continuous development of critical care, severe cardiogenic shock is still associated with high mortality [6–9]. Although an increasing number of studies, with a larger number of patients, report on adverse events associated with ECMO support, the approximate rates of complications are still very heterogeneous, in part because of small study populations [10]. Multiple studies strived to evaluate the potential benefit of ECMO support, but due to methodological issues, its efficacy remains controversial [11–13].

Given the above, our study aims to summarize the evidence and provide a comprehensive review of va-ECMO support outcomes in adult patients with refractory cardiogenic shock. We conducted a meta-analysis to examine mortality and complication rates in published studies, and we provide a summary of the demographic and clinical characteristics of critically ill patients undergoing ECMO support.

## Materials and methods

We conducted a systematic review and meta-analysis of all studies reporting on va-ECMO support, complying with the PRISMA (Preferred Reporting Items for Systematic Reviews and Meta-Analyses) guidelines (Additional file 1: Table S1) [14]. This study is registered in the International prospective register of systematic reviews (PROSPERO) under number CRD42022326365.

The primary endpoint was the estimation of in-hospital mortality associated with the use of va-ECMO in patients with cardiogenic shock. Secondary endpoints included analysis of data on individual adverse events and mortality (in-hospital mortality, brain death, and death during ECMO support). The included studies comprised patients who underwent va-ECMO support, reporting on the incidence of adverse events and mortality. As our review and meta-analysis primarily aims at describing patient outcomes, we did not cover any potential comparators to the applied interventions, Additional file 1: Table S2.

### Search

A systematic literature search was performed in Medline (PubMed) and Scopus (Elsevier) databases (data range up to May 1, 2022), using the combination of the following terms: ECMO, ECLS, ELS, extracorporeal, membrane, oxygen, life, support; fatal, death, mortality; complications and adverse (Additional file 1: Table S3). To ensure completeness of the search, we also searched the reference lists of the included studies, gray literature, and Google Scholar. In case of the full text of the study being not available, the authors of the studies were contacted. We included all studies reporting on (1) va-ECMO only, (2) both adverse events and in-hospital mortality, and (3) more than 100 patients with the patient follow-up to discharge from the hospital. Excluded were (1) all studies reporting on less than 100 patients, (2) reporting selectively on patients under 16 years, and (3) duplicate publications. Furthermore, we excluded studies with the main focus on extracorporeal support as a bridge to transplantation and durable mechanical circulatory support (e.g., ventricular assist device) or extracorporeal cardiopulmonary resuscitation (eCPR) as these groups of patients’ present extremes in means of patient outcome. To avoid overlapping of patients with original studies, systematic reviews and meta-analyses were excluded. Studies reporting on the results from the same institutions or ELSO registry were also excluded, as the overlapping of patients with the submitting center could not be excluded. Finally, we excluded studies with a main focus other than cardiogenic shock (i.e., transport of ECMO patients), or reported in other languages than English. Based on the methodology used in prior systematic reviews and meta-analyses, we chose a study sample size cutoff of 100 patients, to exclude the influence of case reports and small studies [15]. All study and data restrictions are presented in Additional file 1: Table S2.

The title and abstract screening were performed by 2 independent assessors (SR, DJ). Full-text articles of selected studies were reviewed and included if they met the inclusion eligibility criteria. In case of insufficient clarity in data presentation and presumable unreliable information, the study was excluded from the analysis. Any potential conflict in study selection was solved by reaching consensus in the research team.

### Data extraction and synthesis

Two authors (SR, DJ) independently extracted relevant data regarding the basic study characteristics, patient demographics, reported complications, and mortality including the ECMO support technical information. Detailed information on the data extraction and synthesis is available in Additional file 1: Table S4. The definitions for reported outcomes were the ones adopted by the investigators of the included studies.

For comparison and to standardize the results of included studies, we performed simple calculations: (1) in case if only the female sex was reported, the number of male patients was calculated from the total number of patients, (2) percentage was computed into original values and original values into percentage where needed, (3) in case if outcome reported for compared groups, the overall sum was computed, and (4) ECMO support duration reported in hours was computed into days. All calculations were performed by 2 authors (SR, CO) independently.

### Quality assessment

The methodological quality of studies was evaluated with the Newcastle–Ottawa scale for assessing the quality of nonrandomized studies in meta-analyses [16]. A study was considered to be of good quality if scored with 7 out of 9 Newcastle–Ottawa scale stars, fair if it achieved 5, and low-quality with less than 5 stars (Table 1). Two authors (SR, CO) independently evaluated the methodological quality of the studies; disagreements were resolved through consensus within the research team.

**Table 1:** Characteristics of 32 included studies (n = 12,756)

Authors	Country	Study period	Prospective study	Patients	Age (years)	BMI (kg/m^2^)	ECMO duration (days)	Pre-ECMO cardiac arrest	Population	Patient groups	NOS
Aso et al. [21]	Japan	2010–2013	No	4658	64.8			2187	Cardiogenic shock	None^b^	Good
Aubin et al. [54]	Germany	2011–2015	No	160	56		4^a^	102	Refractory circulatory failure or cardiac arrest	Survivors and non-survivors	Fair
Bonacchi et al. [38]	Multicentric	2004–2018	Yes	209	67.5	25.8	5.3	13	Postcardiotomy cardiogenic shock	Survivors and non-survivors	Fair
Cakici et al. [35]	Turkey	2010–2015	No	148	56.6	25.2	5^a^		Cardiogenic shock	Distal perfusion catheter and arterial side-graft technique	Good
Choi et al. [55]	Korea	2004–2018	No	253				181	AMI patients with cardiogenic shock	ECMO used before and after revascularization, and ECPR	Good
Elsharkawy et al. [56]	USA	1995–2005	No	233					Postcardiotomy patients	Survivors and non-survivors	Fair
Fux et al. [57]	Sweden	2006–2015	No	105	62^a^	26.2^a^	7^a^	31	Refractory postcardiotomy cardiogenic shock	Survivors and non-survivors	Good
Karatolios et al. [58]	Germany	2014–2019	No	123	61.25	28.42	5.4^a^	72	Cardiogenic shock due to AMI, dilated cardiomyopathy, or myocarditis	Impella and va-ECMO^c^	Good
Laimoud et al. [39]	Saudi Arabia	2015–2019	No	106	40.2	26.5			Cardiogenic shock	Survivors and non-survivors	Good
Lan et al. [59]	Taiwan	1994–2008	No	607	53.8				Postcardiotomy cardiogenic shock, acute myocarditis, cardiomyopathy, AMI, and acute rejection after heart transplantation	Survivors and non-survivors	Good
Li et al. [60]	China	2011–2012	No	123	56.2		4.4		Postcardiotomy cardiogenic shock	Survivors and non-survivors	Good
Liao et al. [24]	China	2008–2019	No	179			4.8	54	Low cardiac output after open-heart surgery, AMI, cardiomyopathy, pulmonary embolism, myocarditis	Presence of lower limb ischemia	Good
Liem et al. [40]	USA	2010–2018	No	102	52		11.2		Cardiogenic shock	ECPR and cardiogenic shock^b^	Fair
Loforte et al. [25]	Italy	2006–2012	No	228	58.3		10.9	29	Postcardiotomy cardiogenic shock	Postcardiotomy, donor graft failure, AMI, heart failure, myocarditis	Good
Lunz et al. [22]	Germany	2010–2016	No	223	58.1^a^	26.1^a^	3^a^	146	Femoral Va-ECMO approach for low cardiac output, cardiac failure during coronary intervention and resuscitation	Presence of limb ischemia	Fair
Masha et al. [36]	USA	2012–2016	No	223				144	Cardiac arrest, cardiogenic shock, AMI, support of heart transplant or left ventricular assist device	Survivors and non-survivors	Good
Mazzeffi et al. [61]	USA	2010–2015	No	121					Postcardiotomy shock, other cardiogenic shock, or respiratory failure with cardiac dysfunction	ACT and aPTT	Good
McCloskey et al. [62]	USA	2000–2017	No	187	47	29.7	3.1^a^		Failed weaning from CPB, AMI, refractory arrhythmia, pulmonary embolism, decompensated heart failure and others	Survivors and non-survivors	Good
Papadopoulos et al. [63]	Germany	2001–2013	No	360	62		7	50	Postcardiotomy cardiogenic shock	None	Fair
Radakovic et al. [64]	Germany	2010–2019	No	158					Postcardiotomy shock, cardiac arrest, failed weaning from CPB, refractory cardiogenic shock, right-heart failure, and left-heart failure	Central and peripheral cannulation	Good
Rastan et al. [65]	Germany	1996–2008	No	517	63.5		3.3	32	Postcardiotomy cardiogenic shock	Survivors and non-survivors	Fair
Ro et al. [66]	Korea	2005–2012	No	253	58.8		3^a^	80	Cardiogenic shock	Presence of IABP	Good
Roth et al. [67]	Germany	2011–2018	No	344	59		7.6		Cardiogenic shock	Presence of thromboembolic complications	Good
Rubino et al. [37]	UK	2008–2016	No	101	57.1		5^a^		Va-ECMO after cardiac surgery	Survivors and non-survivors	Good
Salna et al. [68]	USA	2008–2019	No	431	60^a^	28.3^a^	4.5^a^	42	Postcardiotomy shock, AMI, acute decompensated heart failure, ECPR, primary graft dysfunction, unstable arrhythmia, pulmonary embolism, and others	Body mass index categories	Good
Son et al. [69]	USA	2014–2018	No	105	54.9		5.6	48	Cardiogenic shock or ECPR	Presence of acute limb ischemia	Good
Toivonen et al. [70]	Multicentric	2010–2018	No	781					Postcardiotomy cardiogenic shock	Presence of neurologic injury	Good
Vigneshwar et al. [71]	Multicentric	2013–2018	No	789	54.8	29.8	4.3^a^	119	Postcardiotomy shock, cardiomyopathy, cardiac arrest, AMI, and acute rejection of heart transplant	Survivors and non-survivors	Good
Wood et al. [72]	USA	2011–2018	No	203					Cardiogenic shock, postcardiotomy shock, ECPR, acute pulmonary embolism and sepsis	Presence of anticoagulation	Good
Wu et al. [73]	Taiwan	2003–2009	No	110	60		6		Postcardiotomy cardiogenic shock	Survivors and non-survivors	Fair
Yau et al. [74]	USA	2011–2016	No	154	55		3.7^a^		Patients with femoral va-ECMO	None	Good
Zhigalov et al. [23]	Germany	2009–2019	No	462	66.2	27.9	4^a^	115	Cardiogenic shock or ECPR	Presence of postcardiotomy	Good

^a^Median value. NOS: Newcastle–Ottawa scale score, AMI: acute myocardial infarction, va-ECMO: venoarterial extracorporeal membrane oxygenation, ECPR: extracorporeal cardiopulmonary reanimation, CPB: cardiopulmonary bypass, ACT: activated clotting time, aPTT: activated partial thromboplastin time, IABP: intra-aortic balloon pump

^b^Authors included only the group of patients with cardiogenic shock in the analysis

^c^Authors included only the group of patients with VA-ECMO in the analysis

### Statistical assessment

Statistical analysis and visualizations were performed with “meta”, “metafor” and “dmetar” packages of R software environment version 4.0.0 (R Core Team 2020: R: A language and environment for statistical computing. R Foundation for Statistical Computing, Vienna, Austria). For the pooling of single proportions, we used the inverse variance methods with logit transformation. Confidence intervals for individual studies were estimated with Clopper–Pearson method. The heterogeneity between-study and its possible causes were explored by Cochran’s Q test and *t*^*2*^ statistics, the Baujat plot, graphic display of heterogeneity analysis—GOSH [17, 18], and quantified with the *I*^*2*^ statistic. The univariate meta-regression analysis was used to identify potential predictors. Further subgroup analyses were performed to evaluate whether the prespecified study characteristics could account for the overall in-hospital mortality (i.e., prospective, or retrospective data collection, study setting, geographical region, publication year, period, and duration of data collection, reporting on less or more of 200 patients, the proportion of male patients, including of ECPR patients and their fraction). Publication bias was assessed using trim and fill [19], contour-enhanced funnel plot [20], and Egger’s test. To confirm the consistency of the main analysis, the sensitivity analysis was performed by excluding the potential effect of influence study on the results of the meta-analysis. A significance level of 0.05 was applied.

## Results

### Search results and description of studies

**Fig. 1:**
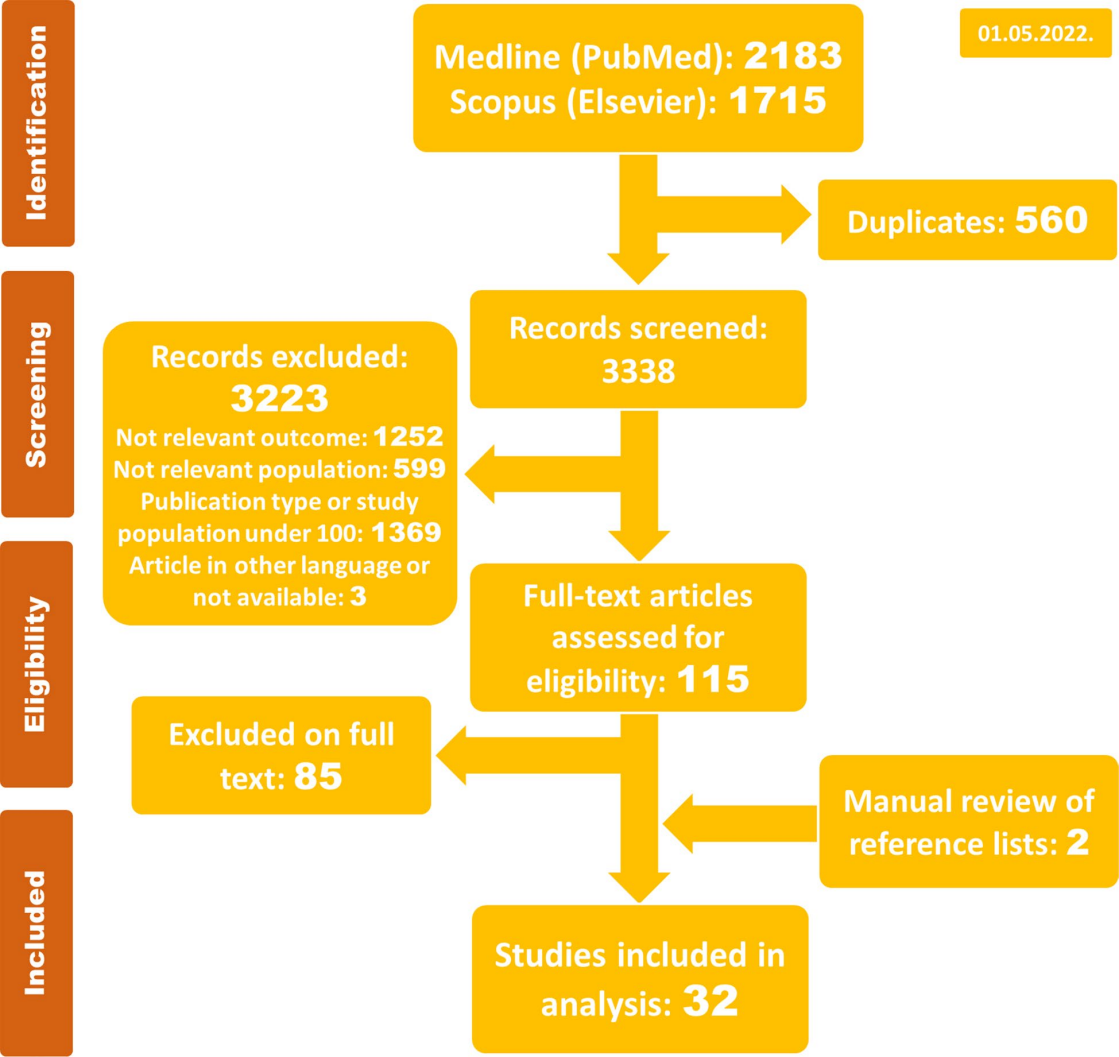
Preferred reporting items for systematic reviews and meta-analyses (PRISMA) flow chart of the search process

The systematic search yielded 2183 references in Medline via PubMed and 1715 in Scopus (Elsevier) database (May 1, 2022). After duplicates removal, a total of 3338 articles were selected for the titles and abstracts screening. In a second step, 3223 papers were excluded: 1369 due to publication type or patient population under 100, 599 addressed populations not relevant for the present analysis, and 1252 addressed irrelevant outcomes (Additional file 1: Table S2). A flow chart of the search process is illustrated in Fig. 1. The main excluded studies are presented in Additional file 1: Table S5. Thereby, 115 publications were selected for full-text screening, of which 85 were excluded once they reported a non-relevant outcome or used the same or similar patient data as other publications. Finally, our systematic assessment of studies comprised 32 publications, including 2 publications retrieved from the manual search of references [21, 22].

The main features of the included studies are presented in Table 1. Analyzed articles reflect the situation from USA (*n* = 9), Germany (*n* = 8), Taiwan (*n* = 2), Korea (*n* = 2), China (*n* = 2), Italy (*n* = 1), Japan (*n* = 1), Saudi Arabia (*n* = 1), Sweden (*n* = 1), Turkey (*n* = 1) and United Kingdom (*n* = 1), and three multicentric publications. Twenty-five of the 32 studies reported on in-hospital mortality and more than 2 complications, while 7 addressed 2 or fewer complications. Only one study used the ELSO definition of bleeding [23]. The reviewed publications obtained the data on ECMO support outcomes mostly through prospective or retrospective databases, hospital records, administrative claims databases, and local or national registers. Finally, the methodological quality assessment of studies showed no low-quality study, 8 studies were rated as fair, and 24 as being of good quality (Table 1).

### Patient population and outcomes

In the period from 1994 to 2019, a total of 12,756 patients received va-ECMO due to refractory cardiogenic shock. The analyzed population comprised of 69% male adult patients, with a pooled mean age of 61.1 years and a mean BMI of 28.2 kg/m^2^. ECMO support was employed for an average of 5.3 days. In total 3421 (pooled 30.2%) patients experienced cardiac arrest before or during ECMO support implantation, and only two studies [22, 24] reported on SOFA score (ranging from 11 to 12.5) and one on SAPS II score [25].

In total 8493 of 12,756 patients died during hospital stay. More than one-third of patients died during ECMO support (37.4% of all and 53.2% of all deceased patients). In-hospital mortality ranged from 40% to 75%, and 154 patients were diagnosed with brain death (pooled 13.1%, [95% CI 9.4; 17.8]), Additional file 1: Table S6.

The need for renal replacement therapy and the presence of limb ischemia were the most often reported adverse events (23 studies), followed by any bleeding and death on-support (15 and 14 studies, respectively), Table 2 and Additional file 1: Table S6.

**Table 2:** Reporting of ECMO-related outcomes and complication rate

Outcome	Number of studies reporting data (events/population)	Pooled rate (95%CI)	*I*^*2*^ (*p* value)	Reported range (%)
Mortality				
Brain death	4 (154/1042)	13.1 (9.4; 17.8)	65% (0.035)	7.6–16.6
Death during ECMO	14 (2909/7783)	42.9 (38.3; 47.7)	91% (< 0.001)	33.4–53.5
In-hospital mortality	32 (8493/12756)	62.2 (58.8; 65.5)	92% (< 0.001)	40.3–75.2
Stroke				
CNS complications (not specified)	7 (360/2450)	12.5 (13.9; 16.8)	82% (< 0.001)	5.4–19.1
Cerebral bleeding/hemorrhagic stroke	12 (263/3969)	5.6 (3.4; 9.0)	92% (< 0.001)	2.7–25.4
Ischemic stroke	13 (414/4371)	9.8 (7.2; 13.1)	89% (< 0.001)	1.4–26.9
Stroke (not otherwise specified)	10 (206/2704)	8.6 (5.3; 13.5)	91% (< 0.001)	2.3–25.5
Renal failure				
Renal failure	7 (693/1351)	50.5 (31.7; 69.2)	97% (< 0.001)	9.5–85.7
Renal replacement therapy	23 (4879/11186)	44.3 (39.2; 49.5)	96% (< 0.001)	10.3–70.5
Infections				
Infection (not otherwise specified)	4 (290/1395)	18.8 (14.3; 24.2)	80% (0.002)	13.0–24.7
Pneumonia	8 (617/2298)	23.7 (16.2; 33.3)	95% (< 0.001)	7.9–61.0
Sepsis	9 (489/2529)	17.8 (14.3; 21.9)	83% (< 0.001)	6.4–28.2
MODS	3 (171/584)	24.4 (13.3; 40.4)	93% (< 0.001)	8.9–37.3
Bleeding				
Any bleeding	15 (1971/4523)	48.5 (40.6; 56.4)	96% (< 0.001)	17.8–97.0
Surgical site bleeding	2 (66/390)	14.9 (3.7; 44.4)	96% (< 0.001)	7.4–27.3
Cannulation site bleeding	8 (224/1464)	16.8 (11.1; 24.7)	91% (< 0.001)	3.9–28.4
Gastrointestinal bleeding	6 (74/1335)	5.9 (3.3; 10.5)	84% (< 0.001)	0.9–12.3
Cardiac tamponade	3 (42/406)	10.5 (7.9; 13.9)	0% (0.423)	8.4–12.7
Pulmonary hemorrhage	2 (24/305)	8.4 (3.5; 18.7)	79% (0.029)	5.4–12.7
Disseminated intravascular coagulation	1 (26/148)	–	–	–
Hemolysis	1 (6/462)	–	–	–
Thrombosis				
Any thrombosis	5 (187/921)	13.4 (6.7; 25.1)	93% (< 0.001)	2.0–34.0
Limb ischemia	23 (868/5932)	12.2 (8.7; 16.9)	96% (< 0.001)	3.7–43.9
Limb amputation	5 (19/1368)	1.5 (1.0; 2.3)	0% (0.733)	0.4–1.9
Arterial thrombosis	1 (63/344)	–	–	–
Venous thrombosis	1 (11/344)	–	–	–
Pulmonary embolism	1 (3/203)	–	–	–
Mechanical complications				
Circuit component clots	3 (12/771)	1.8 (0.5; 6.7)	81% (0.006)	0.9–5.7
Oxygenator replacements	4 (111/1253)	9.0 (3.7; 20.3)	92% (< 0.001)	0.0–25.6

*ECMO* Extracorporeal membrane oxygenation, *CNS* Central nervous system, *MODS* Multiple organ dysfunction syndrome

Renal failure and any kind of bleeding, where the most often experienced adverse events (pooled 50.5% [95%CI 31.7; 69.2] and 48.5% [95%CI 40.6; 56.4], respectively), followed by the need for renal replacement therapy (44.3% [95%CI 39.2; 49.5]). However, differentiation of bleeding severity was impossible due to distinct variations in definitions, from any bleeding to bleeding requiring reoperation and blood product transfusion. Surgical and cannulation site bleeding were reported with 14.9% (95%CI 3.7; 44.4) and 16.8% (95%CI 11.1; 24.7), respectively. Thrombosis, as reported in five studies, had a pooled prevalence of 13.4% (95%CI 6.7; 25.1). Finally, pneumonia and sepsis had an incidence of 23.7% (95%CI 16.2; 33.3) and 17.8% (95%CI 14.3; 21.9), respectively (Table 2 and Additional file 1: Table S4).

Neurological complications were reported in more than half of the studies, with cerebral bleeding occurring in 5.6% (95%CI 3.4; 9.0) and ischemic stroke in 9.8% (95%CI 7.2; 13.1) of patients. The type of stroke was not specified in ten studies (pooled 8.6% [95%CI 5.3; 13.5]) and not-specified neurological complications were reported in 12.5% (95%CI 13.9; 16.8) of patients.

Regarding mechanical ECMO data, a centrifugal pump was used in all 11 studies, and 9 studies reported the use of UFH coated circuits. Information on the anticoagulation regime was provided in 17 studies (UFH), with the target ACT ranging from 140 to 220, and aPTT from 40 to 80 s, see Additional file 1: Table S7. None of the studies reported on the use of argatroban or other types of anticoagulation as primary anticoagulation strategy. Mechanical complications were rather seldom reported and affected 123 of 1821 patients, with most of them requiring oxygenator replacement (111 patients), Table 2.

**Fig. 2:**
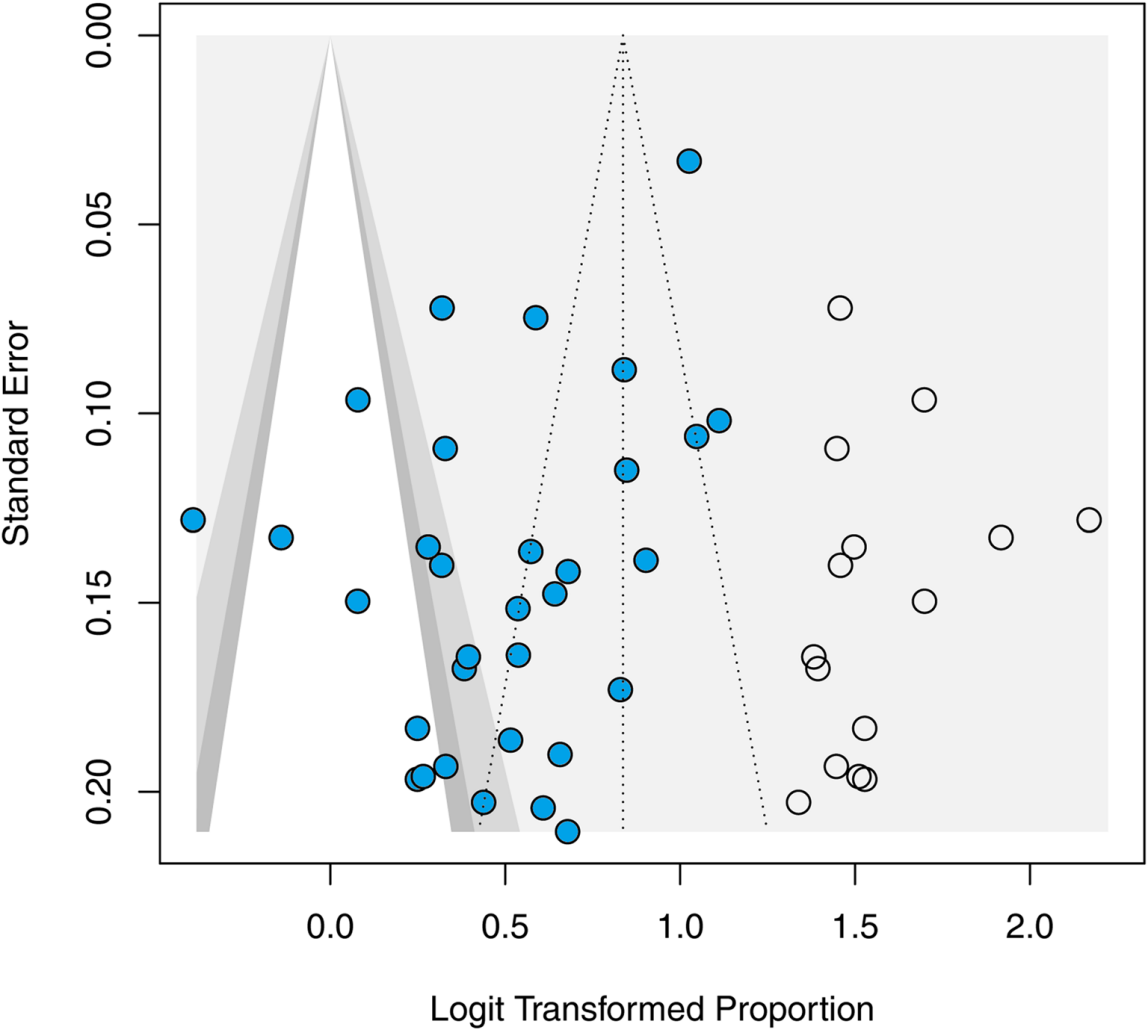
Funnel plot with the trim-and-fill method. Solid circles present the analysis of included studies. Open circles indicate missing studies imputed by the trim-and-fill method

The publication bias of the studies included in the meta-analysis was confirmed using a funnel plot (Fig. 2), and with the linear regression test of funnel plot asymmetry (*p* = 0.021).

**Fig. 3:**
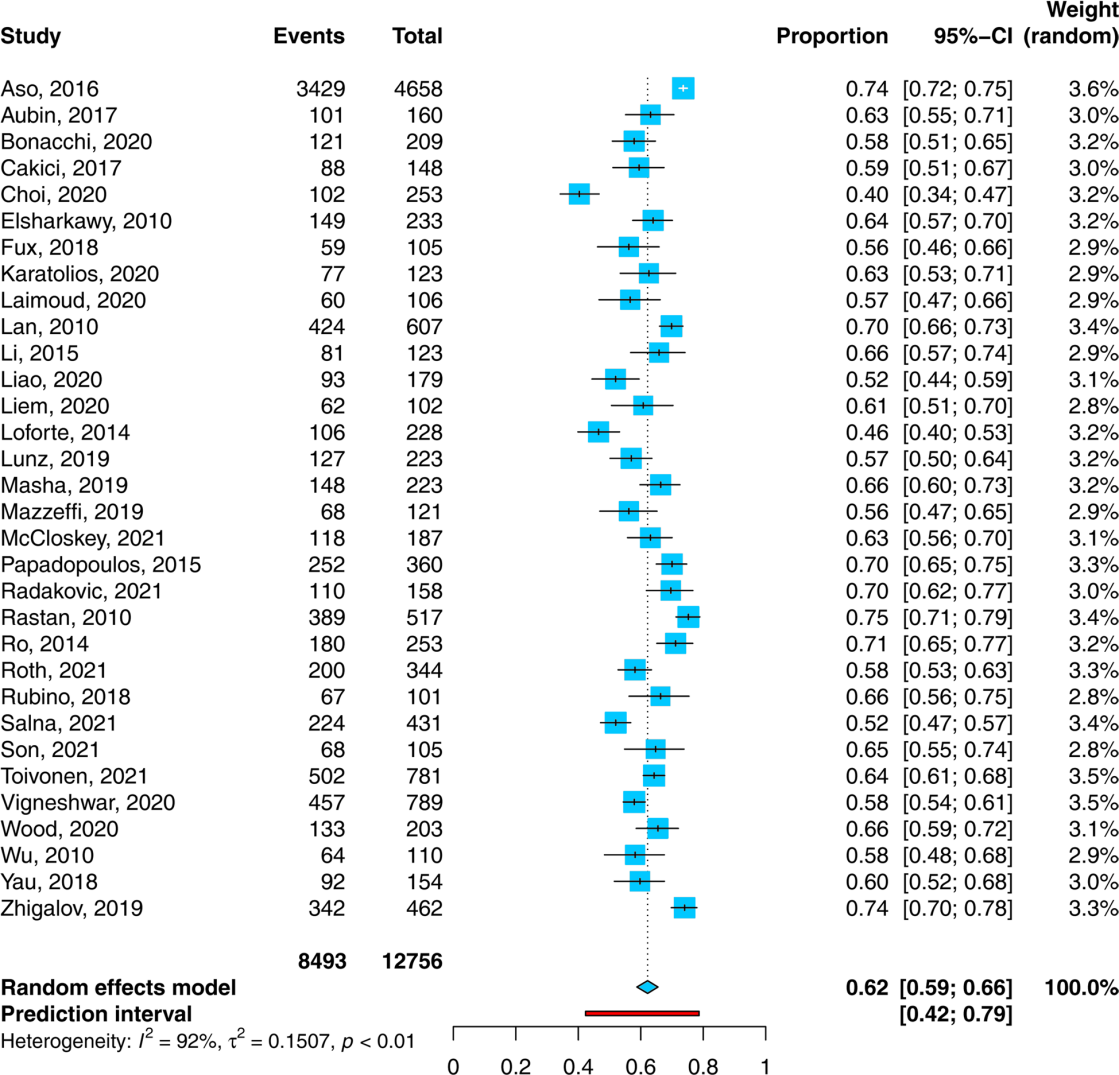
Forest plot: proportion of non-survivors among cardiogenic shock patients requiring extracorporeal membrane oxygenation support

Pooled in-hospital mortality of patients with cardiogenic shock receiving ECMO support (32 studies, 12,756 patients) was 62.2% (95% CI 58.8; 65.5) with a heterogeneity of *I*^*2*^ = 92% (95%CI 89.9; 93.8; Q = 391.3, Tau^2^ = 0.151, *p* < 0.001), Fig. 3. Influence analysis revealed one influential study [21], Additional file 1: Figure S1. After its exclusion, the remaining 31 studies had 8098 patients with 5064 events and the pooled in-hospital mortality did not change (62%, 95%CI 59; 65, *I*^*2*^ = 87%), Additional file 1: Table S8. Subgroup analyses did not show a significant influence of analyzed parameters on the overall in-hospital mortality (Additional file 1: Table S9).

Univariable meta-regression analyses identified age over 60 years (*n* = 23; b = 0.333; *p* = 0.013), shorter ECMO duration (*n* = 22; *b* = − 0.066; *p* = 0.048) and presence of infection (*n* = 4; *b* = 0.033; *p* = 0.017) being associated with in-hospital mortality. The cannulation site bleeding (*n* = 8; b = − 0.029; *p* < 0.001) was associated with reduced in-hospital mortality, Additional file 1: Table S9.

## Discussion

The present systematic review and meta-analysis investigated the use of va-ECMO support in patients with refractory cardiogenic shock, including 32 studies with 12,756 patients. Our work presents the largest up-to-date analysis on va-ECMO outcomes in adult patients. We found a pooled in-hospital mortality as high as 62.2%, a slightly higher than reported currently by ELSO [5]. Moreover, we demonstrated an age over 60 years, a shorter ECMO duration and the presence of an infection being associated with an increased in-hospital mortality.

### ECMO support and all-cause in-hospital mortality

Despite several randomized controlled trials being under way (ECLS-SHOCK-NCT03637205; EUROSHOCK-NCT03813134; ECMO-CS-NCT02301819; and ANCHOR-NCT04184635), the current evidence is based mostly on retrospective studies, systematic reviews, and meta-analyses. Moreover, previous meta-analyses included a rather smaller number of patients and reported on significant heterogeneity in the results [7, 26]. A meta-analysis from 2015 reported an in-hospital survival of 40.2% for patients receiving va-ECMO in cardiogenic shock and cardiac arrest (16 studies with 841 patients) [26]. The complication rates were particularly high for renal impairment (47.4%), infection (25.1%) and neurologic deficits (13.3%) [26]. More recently, another meta-analysis on outcomes of va-ECMO for refractory cardiogenic shock (5292 patients) reported a 43.0% in-hospital, a 36.7% 1-year, and a 29.9% 5-year survival [7]. At a first glance, our in-hospital mortality of 62% seems higher than the above-mentioned works. However, the current ELSO International Summary of Statistics report, including 50,371 ECMO runs in adults, reported a comparable 58.2% in-hospital mortality [5]. The on-support mortality was comparable as well (44.4% vs. 43% in our work). Furthermore, a recent ELSO database analysis of the association between mechanical unloading and va-ECMO outcomes (12,734 patients, mean age 53.7 years, median ECMO duration 4 days) reported an on-support and an in-hospital mortality of 46.2% and 58.6%, respectively [27]. The discrepancy of in-hospital mortality to our work may be partly explained by the selection of patients with peripheral cannulation only, their age, or the limitations inherent to the ELSO registry itself. Therefore, with our large data set, we were able to confirm the earlier observed high mortality.

Nearly one-third of the patients (pooled 30.2%, 95% CI 21.47; 40.7) experienced cardiac arrest before or during ECMO implantation. Surprisingly, cardiac arrest did not change mortality, which is a rather encouraging finding (Additional file 1: Table S9). These findings should support clinicians to indicate ECPR in well-selected patients and warrants the further emergence of ECPR.

Regardless of decades of research, the efficacy of ECMO support in cardiogenic shock has still to be proven. A retrospective trial from the US revealed a low mortality rate of 49% in about 800 ECMO runs in patients experiencing cardiogenic shock [11]. Surprisingly, in a matched cohort the mortality rate of patients without ECMO was as low as 4%. However, this study is limited by matching methods and missing the variables describing the clinical severity in both groups. It could be that the patients receiving ECMO support were simply sicker. This is in contrast to the recent ARREST trial, where Yannopoulous et al. observed a strikingly higher rate of survival to hospital discharge with va-ECMO support in an ongoing cardiac arrest compared to the standard advanced life support (43% vs. 7%) [28]. Whether such a benefit or otherwise of va-ECMO therapy compared to the standard of care is possible in cardiogenic shock (without cardiac arrest) will be evaluated in the ongoing prospective randomized trials EURO-SHOCK and ECLS-SHOCK.

Furthermore, a recent international survey from 60 countries demonstrated various therapy approaches in cardiogenic shock [29]. In the case of acute myocardial infarction, about 42% use percutaneous coronary intervention (PCI) if an electrocardiogram is suggestive of ischemia, one-third perform PCI to all patients in cardiogenic shock, whereas one-fifth only if the universal definition of myocardial infarction criteria are fulfilled. Given these different institutional approaches, the comparison of mechanical cardiac support in cardiogenic shock to therapy without it is hindered. However, the early use of revascularization therapy reduced the initially high mortality rates, but in-hospital mortality remains significant (27–51%) [30]. Despite an overall increase in PCIs, Amsterdam et al. recently even described a potential rise of mortality from 27% to 30% due to increasing patient complexity and care being more often delivered by less experienced lower volume centers [31]. Finally, an early invasive hemodynamic assessment may help the identification of cardiogenic shock phenotype, which is important for further treatment, since distinct etiologies may respond differently to medical and device-based management. Hopefully, the undergoing prospective trials will be able to shed light on these important issues.

### Complications and adverse events during va-ECMO

Current literature comprises mostly smaller studies offering a wide range of reported complications, commonly without any standardization which may be attributed to the presence of different criteria for the identification and reporting of adverse events. However, the pooled rate of adverse events in our data should more accurately reflect the rate of expectable complications.

Comparable to literature, hemorrhage was the second most frequent complication in nearly half of the patients, which is in line with the largest meta-analysis so far, reporting any kind of hemorrhage in 40% [15]. Going into greater detail confirmed the cannulation and surgical area being the most common sites of bleeding [32–34]. However, the latter findings may be weakened by the fact that only one study reported on the use of the ELSO bleeding definition [23], despite the definition´s existence of more than 8 years.

Recently, a study from Turkey demonstrated a need for continuous renal replacement therapy in about one-quarter of 148 patients with refractory cardiogenic shock. In contrast to that, we found rates nearly as twice as high (44.3% vs. 24.4%) representing the most common complication [35]. Moreover, the same group reported renal failure in only 9.5%, which is inconsistent (1) with the higher rate of the continuous renal replacement therapy in the same study and (2) our findings in 1351 patients (renal failure in 50.5%). This may be due to the retrospective nature of the above-mentioned study. Furthermore, these authors used different definitions of renal failure. Masha et al. defined acute renal failure as serum creatinine increase of more than 1.5 mg/dl with or without renal replacement therapy [36]; Rubino et al. as renal impairment requiring continuous renal replacement therapy [37]; and Zhigalov et al. as a new renal dysfunction requiring renal replacement therapy or a rise in serum creatinine (greater than three times baseline or greater than 5 mg/dL) [23]. Other authors defined renal failure as an acute kidney injury or organ dysfunction, failing to provide a more detailed definition [35, 38–40]. Finally, our findings are in line with an earlier meta-analysis on ECMO in cardiogenic shock in nearly three-time smaller patient sample compared to ours [10]. Cheng et al. reported a pooled estimate rate for acute kidney injury of 55.6%, and need for continuous renal replacement therapy of 46.0%, respectively [10]. The marginal alteration in pooled prevalence may be explained by the greater availability of evidence due to increased number of studies, selection of studies with larger patient samples, and an overall sample of patients in the present analysis. Moreover, the potential impact of technological advances and critical care medicine development cannot be completely excluded.

The low rate of thrombosis is most probably due to an underestimation of thrombotic events and lacking of regular radiological investigations or post-mortem examinations in the majority of the predominantly retrospective studies [41].

### Risk factors for mortality

Univariable meta-regression analyses identified age over 60 years, shorter ECMO duration and presence of infection as variables associated with in-hospital mortality. The studies reporting a higher incidence of cannulation site bleeding were unexpectedly associated with a reduced in-hospital mortality.

The role of the age in va-ECMO support of patients with cardiogenic shock remains controversial. According to the ELSO guidelines, there is no defined age cutoff, but an age-related risk should be considered [4, 42]. In the case of a COVID-19-related ECMO indication, ELSO defined an age of more than 65 years as a relative and an even higher age as an absolute contraindication for ECMO initiation [43]. A recent retrospective study pointed out the importance of a patient-oriented and individualized approach in decision-making related to ECMO support initiation, arguing against the use of any age-related cutoffs [44]. In our work, an age of more than 60 years (median age of reported population), was associated with increased mortality (Additional file 1: Figure S2). However, as age alone should not be a risk factor, decision-making should be focused on the severity of the disease in combination with comorbidities, frailty, and rehabilitation potential [44, 45].

We found a shorter ECMO support duration being associated with an increased mortality, which is presumably rather a consequence than a mortality influencing factor itself. Most likely the share of patients with shorter than average ECMO runs will experience the more severe underlying pathologies. A further possible explanation may be the presence of the immortal time bias, as patients dying early on ECMO support may not have had enough time for organ recovery [46]. Moreover, the sickest patients may die anyway, regardless of ECMO support. Finally, despite the different setting in vv-ECMO, these findings are consistent with a meta-analysis reporting on vv-ECMO [47].

Our meta-analysis revealed the presence of infection as another risk factor for increased mortality which is based on the data from only four studies. Even with scant evidence on infection during ECMO support, the ELSO registry analysis reported a prevalence of 10–12% [48–50]. Our higher pooled incidence of 18% may be explained by a still small patient sample and seldom reporting on infections as complications of ECMO support. Clearly, further research should focus on more detailed reporting of infections (local and systemic) and its influence on outcomes.

Finally, studies with lower overall mortality reported a higher incidence of cannulation site bleeding. This is surprising as one would assume the opposite. However, as longer va-ECMO is needed, the possibility of adverse events in general may increase, including cannulation site bleeding. Moreover, the delayed minor bleeding during the ECMO course may be associated with a longer anticoagulation exposure and cumulative risk of hemorrhage. The meta-regression did not identify further factors that could be associated with mortality (Additional file 1: Table S9).

### Strengths and limitations

The strengths of this work include robust inclusion and clear exclusion criteria. Our work included 32 publications with 12756 patients from almost all continents with at least fair quality of data. Moreover, we controlled for potential overlapping of patients within different studies, by excluding the studies from the same institutions and from the ELSO registry. Despite all the benefits international registers might provide, these results may not completely represent the real-life situation worldwide, as notification and selection bias may affect studies based on a big database. The inclusion of patients in the ELSO register is voluntary, and sites participating in the network are not a random sample of all centers utilizing ECMO support, but selected centers which guaranteed their membership by paying the membership fee [51]. Recent research of the Society of Thoracic Surgeons databases suggested that selected participant centers may improve quality and outcomes, simply by the feedback of collected data, consequently increasing institutional awareness and self-examination, making these systematically different from nonparticipant centers [52]. Therefore, our study presents the result of a predefined and systematically conducted search of two large international scientific databases, where authors can make their work available to the widest audience, independent of their status. Finally, this work is reported according to the recommendations of the PRISMA checklist, addressing all 27 items (Additional file 1: Table S1).

Nevertheless, this work has several limitations. The quality of our results is as strong as all included studies, given the retrospective and single-center nature of most of the studies. Publication and retrieval bias may have arisen, as studies may neither be available, nor published in the searched databases. Moreover, we excluded all studies reporting on less than 100 patients, to reduce the influence of case reports and small studies on the overall outcome. The majority of studies reported on adverse events missed to precisely define the outcome of interest, making a comparison between the studies at least complex. The ELSO definition of major and minor bleeding was used in only one study. Moreover, recommendations on reporting on outcomes and adverse events during ECMO are still missing, in contrast to the minimum reporting criteria for cardiopulmonary bypass [53]. The diversity of study questions led to different variable reporting, so well-established scores to evaluate the severity of the underlying disease were rather seldom reported (SOFA score, APACHE II, SAPS II or III). Furthermore, the majority of reported factors included between 6 and 12 data points. This may reduce the strength of the potential association between examined factors and in-hospital mortality.

Heterogeneity is a well-known limitation of observational retrospective studies, and the high heterogeneity levels observed implied the increased variance of analyzed studies. Therefore, the result of our analysis should be interpreted with caution, as the meta-analytic portion of our work may be limited by heterogeneity observed across studies.

Moreover, we cannot clarify if the observed rates of some adverse events reflect the consequence of ECMO itself or the severity of underlying disease independent of ECMO. It is most likely that complications such as cannulation site bleeding, limb ischemia, and amputation are more directly related to ECMO procedure, whereas renal failure, infections, and stroke may be consequences of cardiogenic shock, and joint effects of comorbidities and critical illness.

## Conclusions

Despite the high rates of mortality in refractory cardiogenic shock, ECMO support can prolong the therapeutic window potentially allowing the heart to recover. This large meta-analysis comprising 12756 patients identified a pooled in-hospital mortality of 62%. Furthermore, patient age, presence of infection and shorter ECMO support duration have been shown to be independently associated with an increased in-hospital mortality. Moreover, adverse events during ECMO support are frequent and with potential for permanent injury or death. Renal failure with the need for renal replacement therapy and bleeding occurrence are the complications with the highest incidence. Protocols and techniques must be developed to reduce the rate of adverse events. Furthermore, use of pre-defined reporting criteria on ECMO is warranted. Finally, randomized trials are necessary to demonstrate the effectiveness of va-ECMO in cardiogenic shock.

## Supplementary Information

**Additional file1: Table S1.** Preferred Reporting Items for Systematic review and Meta-Analysis Protocols (PRISMA-P) 2015 checklist: recommended items to address in a systematic review protocol. **Table S2.** PICOS criteria for inclusion and exclusion of publications. **Table S3.** Search strategy. **Table S4.** Detailed information on the data extraction and synthesis. **Table S5.** Main excluded studies. **Table S6.** Reported ECMO adverse events in the included studies (n = 32). **Table S7.** Patient anticoagulation and ECMO characteristics of the included studies (n = 32). **Table S8.** Influence analysis of studies reporting on in-hospital ECMO mortality. **Table S9.** Univariable meta-regression analyses. **Figure S1.** Influence analysis of studies reporting on in-hospital mortality. **Figure S2. **Meta-regression: scattered-plot of the relationship between age and in-hospital mortality.

## Data Availability

All data generated or analyzed during this study are included in this published article and its supplementary information files. The additional data sets used and analyzed during the current study are available from the corresponding author on reasonable request.

## Abbreviations

ACT: Activated clotting time
aPTT: Activated partial thromboplastin time
AMI: Acute myocardial infarction
CNS: Central nervous system
CPB: Cardiopulmonary bypass
ECMO: Extracorporeal membrane oxygenation
ECPR: Extracorporeal cardiopulmonary resuscitation
ELSO: Extracorporeal life support organization
DIC: Disseminated intravascular coagulation
IABP: Intra-aortic balloon pump
MODS: Multiple organ dysfunction syndrome
NOS: Newcastle–Ottawa scale score
va-ECMO: Venoarterial extracorporeal membrane oxygenation
UFH: Unfractionated heparin
