# Supplementary material for: Extracorporeal membrane oxygenation for cardiogenic shock: a meta-analysis of mortality and complications
Source: Ann Intensive Care. 2022 Oct 5;12:93. doi: 10.1186/s13613-022-01067-9 (PMC9532225; doi:10.1186/s13613-022-01067-9)
Supplement: Supplementary file 1 — Additional file1: Table S1. Preferred Reporting Items for Systematic review and Meta-Analysis Protocols (PRISMA-P) 2015 checklist: recommended items to address in a systematic review protocol. Table S2. PICOS criteria for inclusion and exclusion of publications. Table S3. Search strategy. Table S4. Detailed information on the data extraction and synthesis. Table S5. Main excluded studies. Table S6. Reported ECMO adverse events in the included studies (n = 32). Table S7. Patient anticoagulation and ECMO characteristics of the included studies (n = 32). Table S8. Influence analysis of studies reporting on in-hospital ECMO mortality. Table S9. Univariable meta-regression analyses. Figure S1. Influence analysis of studies reporting on in-hospital mortality. Figure S2. Meta-regression: scattered-plot of the relationship between age and in-hospital mortality. [file 13613_2022_1067_MOESM1_ESM.docx]

**Supplementary material**

**Extracorporeal Membrane Oxygenation for Cardiogenic Shock: A Meta-Analysis of Complications and Mortality**

Sasa Rajsic, Benedikt Treml, Dragana Jadzic, Robert Breitkopf, Christoph Oberleitner, Marina Popovic Krneta and Zoran Bukumiric

# Contents

**Table S1.** Preferred Reporting Items for Systematic review and Meta-Analysis Protocols (PRISMA-P) 2015 checklist: recommended items to address in a systematic review protocol

**Table S2.** PICOS criteria for inclusion and exclusion of publications

Table S3. Search strategy

Table S4. Detailed information on the data extraction and synthesis

**Table S5.** Main excluded studies

**Table S6.** Reported ECMO adverse events in the included studies (n = 32)

**Table S7.** Patient anticoagulation and ECMO characteristics of the included studies (n = 32)

**Figure S1.** Influence analysis of studies reporting on in-hospital mortality

**Table S8.** Influence analysis of studies reporting on in-hospital ECMO mortality

**Table S9.** Univariable meta-regression analyses

**Figure S2.** Meta-regression: scattered-plot of the relationship between age and in-hospital mortality

# Table S1. Preferred Reporting Items for Systematic review and Meta-Analysis (PRISMA) 2020 checklist

| **Section and Topic** | **Item #** | **Checklist item** | **Location where item is reported** |
| --- | --- | --- | --- |
| **TITLE** | | |  |
| Title | 1 | Identify the report as a systematic review. | Page 1 |
| **ABSTRACT** | | |  |
| Abstract | 2 | See the PRISMA 2020 for Abstracts checklist. | Page 1 |
| **INTRODUCTION** | | |  |
| Rationale | 3 | Describe the rationale for the review in the context of existing knowledge. | Page 2-3 |
| Objectives | 4 | Provide an explicit statement of the objective(s) or question(s) the review addresses. | Page 2-3 |
| **METHODS** | | |  |
| Eligibility criteria | 5 | Specify the inclusion and exclusion criteria for the review and how studies were grouped for the syntheses. | Page 2-3 |
| Information sources | 6 | Specify all databases, registers, websites, organisations, reference lists and other sources searched or consulted to identify studies. Specify the date when each source was last searched or consulted. | Page 2-3 |
| Search strategy | 7 | Present the full search strategies for all databases, registers and websites, including any filters and limits used. | Page 2, Supplementary |
| Selection process | 8 | Specify the methods used to decide whether a study met the inclusion criteria of the review, including how many reviewers screened each record and each report retrieved, whether they worked independently, and if applicable, details of automation tools used in the process. | Page 2-3 |
| Data collection process | 9 | Specify the methods used to collect data from reports, including how many reviewers collected data from each report, whether they worked independently, any processes for obtaining or confirming data from study investigators, and if applicable, details of automation tools used in the process. | Page 2-3 |
| Data items | 10a | List and define all outcomes for which data were sought. Specify whether all results that were compatible with each outcome domain in each study were sought (e.g. for all measures, time points, analyses), and if not, the methods used to decide which results to collect. | Page 2-3, Supplementary |
|  | 10b | List and define all other variables for which data were sought (e.g. participant and intervention characteristics, funding sources). Describe any assumptions made about any missing or unclear information. | Page 2-3, Supplementary |
| Study risk of bias assessment | 11 | Specify the methods used to assess risk of bias in the included studies, including details of the tool(s) used, how many reviewers assessed each study and whether they worked independently, and if applicable, details of automation tools used in the process. | Page 3 |
| Effect measures | 12 | Specify for each outcome the effect measure(s) (e.g. risk ratio, mean difference) used in the synthesis or presentation of results. | Page 3 |
| Synthesis methods | 13a | Describe the processes used to decide which studies were eligible for each synthesis (e.g. tabulating the study intervention characteristics and comparing against the planned groups for each synthesis (item #5)). | Page 2-3 |
|  | 13b | Describe any methods required to prepare the data for presentation or synthesis, such as handling of missing summary statistics, or data conversions. | Page 2-3 |
|  | 13c | Describe any methods used to tabulate or visually display results of individual studies and syntheses. | Page 2-3, Supplementary |
|  | 13d | Describe any methods used to synthesize results and provide a rationale for the choice(s). If meta-analysis was performed, describe the model(s), method(s) to identify the presence and extent of statistical heterogeneity, and software package(s) used. | Page 2-3 |
|  | 13e | Describe any methods used to explore possible causes of heterogeneity among study results (e.g. subgroup analysis, meta-regression). | Page 3, Supplementary |
|  | 13f | Describe any sensitivity analyses conducted to assess robustness of the synthesized results. | Page 3 |
| Reporting bias assessment | 14 | Describe any methods used to assess risk of bias due to missing results in a synthesis (arising from reporting biases). | Page 3 |
| Certainty assessment | 15 | Describe any methods used to assess certainty (or confidence) in the body of evidence for an outcome. | Page 3 |
| **RESULTS** | | |  |
| Study selection | 16a | Describe the results of the search and selection process, from the number of records identified in the search to the number of studies included in the review, ideally using a flow diagram. | Page 3, 7 |
|  | 16b | Cite studies that might appear to meet the inclusion criteria, but which were excluded, and explain why they were excluded. | Supplementary |
| Study characteristics | 17 | Cite each included study and present its characteristics. | Table 1 |
| Risk of bias in studies | 18 | Present assessments of risk of bias for each included study. | Table 1 |
| Results of individual studies | 19 | For all outcomes, present, for each study: (a) summary statistics for each group (where appropriate) and (b) an effect estimate and its precision (e.g. confidence/credible interval), ideally using structured tables or plots. | Table 1, Supplementary |
| Results of syntheses | 20a | For each synthesis, briefly summarise the characteristics and risk of bias among contributing studies. | Table 1, Supplementary |
|  | 20b | Present results of all statistical syntheses conducted. If meta-analysis was done, present for each the summary estimate and its precision (e.g. confidence/credible interval) and measures of statistical heterogeneity. If comparing groups, describe the direction of the effect. | Table 1, Supplementary |
|  | 20c | Present results of all investigations of possible causes of heterogeneity among study results. | Page 8-9 Supplementary |
|  | 20d | Present results of all sensitivity analyses conducted to assess the robustness of the synthesized results. | Supplementary |
| Reporting biases | 21 | Present assessments of risk of bias due to missing results (arising from reporting biases) for each synthesis assessed. | Page 8-9  Supplementary |
| Certainty of evidence | 22 | Present assessments of certainty (or confidence) in the body of evidence for each outcome assessed. | Supplementary |
| **DISCUSSION** | | |  |
| Discussion | 23a | Provide a general interpretation of the results in the context of other evidence. | Page 9-13 |
|  | 23b | Discuss any limitations of the evidence included in the review. | Page 9-13 |
|  | 23c | Discuss any limitations of the review processes used. | Page 9-13 |
|  | 23d | Discuss implications of the results for practice, policy, and future research. | Page 9-13 |
| **OTHER INFORMATION** | | |  |
| Registration and protocol | 24a | Provide registration information for the review, including register name and registration number, or state that the review was not registered. | Page 2 |
|  | 24b | Indicate where the review protocol can be accessed, or state that a protocol was not prepared. | Page 2 |
|  | 24c | Describe and explain any amendments to information provided at registration or in the protocol. | Page 2 |
| Support | 25 | Describe sources of financial or non-financial support for the review, and the role of the funders or sponsors in the review. | Page 13 |
| Competing interests | 26 | Declare any competing interests of review authors. | Page 14 |
| Availability of data, code and other materials | 27 | Report which of the following are publicly available and where they can be found: template data collection forms; data extracted from included studies; data used for all analyses; analytic code; any other materials used in the review. | Page 14 |

*From: Page MJ, McKenzie JE, Bossuyt PM, Boutron I, Hoffmann TC, Mulrow CD, et al. The PRISMA 2020 statement: an updated guideline for reporting systematic reviews. BMJ 2021;372:n71. doi: 10.1136/bmj.n71.*

# Table S2. PICOS criteria for inclusion and exclusion of publications

| **Parameter** | **Inclusion criteria** | **Exclusion criteria** |
| --- | --- | --- |
| Population | - Patients older than 16 years | - Patients younger than 16 years |
| Intervention | - Venoarterial ECMO due to cardiogenic shock | - Other forms of ECMO support,  - Main focus on indications other than cardiogenic shock,  - Multiple runs of ECMO support,  - Main focus on use of ECMO as bridge to transplantation and durable mechanical circulatory support or extracorporeal cardiopulmonary resuscitation,  - Inclusion of patients with respiratory failure,  - Transport of ECMO patients,  - Reporting on ECMO support use shorter than six hours,  - Studies excluding deceased patients within first 24h of ECMO initiation,  - ELSO registry studies due to potential of patient overlapping with submitting center  - Studies with the risk of patient data overlapping (we included the study with larger number of patients) |
| Comparator | None | None |
| Outcomes | Primary:  - In-hospital mortality,  - Reported adverse events  Secondary:  - Demographic and clinical characteristics of patients undergoing va-ECMO,  - Individual adverse events and mortality (in-hospital, brain death, death during ECMO support) | - |
| Study design | - Studies reporting on 100 or more patients,  - Clinical randomized trials,  - Controlled before-and-after studies,  - Prospective and retrospective cohort studies,  - Cross-sectional studies  - Grey literature | - Studies reporting on less than 100 patients,  - Publications with the same or overlapping patient population,  - Systematic reviews,  - Meta-analyses,  - Case reports,  - Conference abstracts,  - Letters |
| PICOS, patients/population, intervention, comparator, outcomes, study design; ECMO, extracorporeal membrane oxygenation; ELSO, Extracorporeal Life Support Organization; va-ECMO, Venoarterial extracorporeal membrane oxygenation | | |

# Table S3. Search strategy

| **Database: Medline (PubMed)** |
| --- |
| Date searched: 01.05.2022.  Records retrieved: 2183  Code: (ecmo OR ecls OR els OR extracorporeal AND ((membrane AND oxygen) OR (life AND support))) AND (fatal OR death OR mortality) AND (complications OR adverse) |
| **Database: Scopus (Elsevier)** |
| Date searched: 01.05.2022.  Records retrieved: 1715  Code: TITLE-ABS-KEY ( ecmo OR ecls OR els OR extracorporeal AND ( ( membrane AND oxygen ) OR ( life AND support ) ) ) AND ( fatal OR death OR mortality ) AND ( complications OR adverse ) AND ( LIMIT-TO ( SUBJAREA , "MEDI" ) ) AND ( LIMIT-TO ( DOCTYPE , "ar" ) ) AND ( LIMIT-TO ( EXACTKEYWORD , "Human" ) ) |

# Table S4. Detailed information on the data extraction and synthesis

| **Characteristics** | **Description** |
| --- | --- |
| Basic study characteristics | The name of the first author, publication year, country, center name, study period, study design, number of included patients, main indications for ECMO support, compared groups, name of the register, and follow-up period. |
| Patient demographic characteristics | Sex, age, body mass index (BMI, m/kg^2^), presence of pre-ECMO cardiac arrest, the sequential organ failure assessment (SOFA) score, the acute physiology and chronic health evaluation II (APACHE II) score, simplified acute physiology score (SAPS II and III) score, and ECMO support duration. |
| Adverse events | Total bleeding events, a number of different site bleeding events (surgical and cannulation site, cardiac tamponade, pulmonary hemorrhage, gastrointestinal tract), disseminated intravascular coagulation (DIC), hemolysis, central nervous system complications, hemorrhagic and ischemic stroke (or any stroke if not specified), total thrombotic events (including arterial and venous thrombosis, pulmonary embolism, deep vein thrombosis), limb ischemia, limb amputation, renal failure and renal replacement therapy, pneumothorax, liver dysfunction, presence of pneumonia, sepsis or any infection, and multiple organ dysfunction syndrome. |
| Mortality | Data on brain death, death during ECMO support, intensive care unit (ICU) mortality, and in-hospital mortality. |
| Anticoagulation regime | Type of anticoagulation used, goal activated clotting time (ACT in seconds) or activated partial thromboplastin time (aPTT in seconds). |
| ECMO support technical characteristics | Machine used, pump type, use of unfractionated heparin (UFH) coating; equipment-related adverse events (pump failure, device exchange/replacement, circuit component clots and the number of oxygenator replacements). |

ECMO, extracorporeal membrane oxygenation; BMI, body mass index; ICU, intensive care unit; ACT, activated clotting time; aPTT, activated partial thromboplastin time; UFH, unfractionated heparin; SOFA, sequential organ failure assessment; AOACHE II, acute physiology and chronic health evaluation II; SAPS, simplified acute physiology score; DIC, disseminated intravascular coagulation.

# Table S5. Main excluded studies

| **Study (author and year)** | **Number of patients** | **Reason for exclusion** |
| --- | --- | --- |
| Chen et al. (2017) (1) | 1137 | Risk of patient overlap |
| Chen et al. (2016) (2) | 320 | Risk of patient overlap |
| Chen et al. (2019) (3) | 3251 | Study excludes patients deceased within 24h of ECMO initiation |
| Chung et al. (2020) (4) | 11984 | ELSO registry |
| Coutance et al. (2020) (5) | 415 | Main focus on heart transplantation |
| Dangers et al. (2017) (6) | 105 | Main focus on patients with bridge to transplantation or durable mechanical circulatory support |
| Distelmaier et al. (2020) (7) | 354 | Follow up period not limited on in-hospital |
| Gass et al. (2014) (8) | 135 | Study includes patients with respiratory failure |
| Grandin et al. (2022) (9) | 12734 | ELSO registry |
| Hayanga et al. (2015) (10) | 12458 | Main focus on bridge to transplantation |
| Huckaby et al. (2021) (11) | 13,142 | Main focus on heart transplantation |
| Kashiura et al. (2022) (12) | 847 | Main focus on ECPR |
| Kowalewski et al. (2021) (13) | 7185 | ELSO registry |
| Lee et al. (2016) (14) | 200 | Main focus on ECPR |
| Loforte et al. (2014) (15) | 119 | Not available in English language |
| Loyaga-Rendon et al. (2020) (16) | 19824 | Main focus on bridge to durable mechanical circulatory support |
| Mariscalo et al. (2020) (17) | 719 | Risk of patient overlap |
| Mastoris et al. (2022) (18) | 425 | Main focus on bridge to transplantation or durable mechanical circulatory support |
| Seong et al. (2021) (19) | 496 | Risk of patient overlap |
| Thiagarajan et al. (2009) (20) | 295 | Main focus on ECPR |
| Tsai et al. (2017) (21) | 167 | Study excludes patients deceased within 24h of ECMO initiation |
| Vallabhajosyula et al. (2019) (22) | 2962 | No complications reported |
| Wang et al. (2022) (23) | 12592 | ELSO registry |
| Yeo et al. (2016) (24) | 151 | Follow up not reported |
| Yen et al. (2018) (25) | 139 | Study excludes patients deceased within 24h of ECMO initiation |
| Yu et al. (2011) (26) | 121 | Study includes pediatric patients |
| Yusuff et al. (2021) (27) | 653 | Risk of patient overlap |

ELSO: Extracorporeal Life Support Organization, ECPR: extracorporeal cardiopulmonary resuscitation; ECMO, extracorporeal membrane oxygenation.

# Table S6. Reported ECMO adverse events in the included studies (n = 32)

| **Author** | **Bleeding** | **Surgical site bleeding** | **Cannulation site bleeding** | **Gastrointestinal bleeding** | **Cardiac tamponade** | **Pulmonary hemorrhage** | **CNS complications** | **Hemorrhagic stroke** | **Ischemic stroke** | **Stroke (not defined as bleeding or ischemia)** | **Thrombosis** | **Limb ischemia** | **Limb amputation** | **Renal replacement therapy** | **Renal failure** | **Pneumonia** | **Infection** | **Sepsis** | **MODS** | **Brain death** | **Death during ECMO** | **In-hospital mortality** |
| --- | --- | --- | --- | --- | --- | --- | --- | --- | --- | --- | --- | --- | --- | --- | --- | --- | --- | --- | --- | --- | --- | --- |
| Aso et al. (2016) (28) |  |  |  |  |  |  |  |  |  |  |  |  |  |  | 1765 |  |  |  |  |  | 1554 | 3429 |
| Aubin et al. (2017) (29) | 72 |  |  |  |  |  |  |  | 43 |  |  | 42 |  | 77 |  |  |  | 19 |  |  |  | 101 |
| Bonacchi et al. (2020) (30) | 76 |  |  |  |  |  |  | 53 | 38 | 24 |  | 11 |  | 112 | 134 | 37 |  | 59 |  |  |  | 121 |
| Cakici et al. (2017) (31) |  |  | 27 | 9 |  |  | 8 | 6 | 2 |  | 3 | 6 |  | 36 | 14 | 25 |  | 18 |  |  | 72 | 88 |
| Choi et al. (2020) (32) | 45 |  |  |  |  |  | 28 |  |  |  |  | 23 |  | 98 |  |  |  |  |  |  |  | 102 |
| Elsharkawy et al. (2010) (33) |  |  |  | 15 |  |  | 23 |  |  |  |  |  |  | 101 |  | 59 |  | 48 | 75 |  |  | 149 |
| Fux et al. (2018) (34) |  |  |  |  |  |  |  | 7 | 17 |  | 8 |  | 1 | 74 |  | 64 |  | 25 |  | 8 | 49 | 59 |
| Karatolios et al. (2020) (35) |  |  | 21 |  |  |  |  |  |  | 3 |  | 21 |  |  |  |  |  |  |  |  |  | 77 |
| Laimoud et al. (2020) (36) |  |  |  |  |  |  |  | 13 | 14 | 27 |  |  |  | 50 | 73 |  |  |  |  |  |  | 60 |
| Lan et al. (2010) (37) |  |  |  |  |  |  |  |  |  | 40 |  |  |  | 301 |  |  | 132 |  |  | 101 |  | 424 |
| Li et al. (2015) (38) |  |  |  |  |  |  |  |  |  | 5 |  | 21 |  | 29 |  |  | 16 |  | 11 |  |  | 81 |
| Liao et al. (2020) (39) |  |  | 42 |  |  |  |  |  |  |  |  | 36 |  |  |  |  |  |  |  |  |  | 93 |
| Liem et al. (2020) (40) |  |  | 29 | 12 | 13 | 13 |  | 8 |  | 20 |  | 13 |  |  | 26 | 20 |  |  |  | 9 | 39 | 62 |
| Loforte et al. (2014) (41) | 125 |  |  |  |  |  |  |  |  |  |  | 13 |  | 113 |  |  |  | 43 | 85 | 36 | 84 | 106 |
| Lunz et al. (2019) (42) |  |  |  |  |  |  |  |  |  |  |  | 98 | 1 |  |  |  |  |  |  |  |  | 127 |
| Masha et al. (2019) (43) |  |  |  |  |  |  |  |  |  |  |  |  |  | 147 | 191 |  |  |  |  |  |  | 148 |
| Mazzeffi et al. (2019) (44) | 87 |  |  |  |  |  |  |  |  | 9 | 22 |  |  |  |  |  |  |  |  |  |  | 68 |
| McCloskey et al. (2021) (45) | 108 | 51 | 47 | 9 |  |  |  | 5 | 13 |  |  | 13 |  | 84 |  |  |  |  |  |  |  | 118 |
| Papadopoulos et al. (2015) (46) | 148 |  |  |  |  |  | 43 | 11 | 32 |  |  | 47 |  | 220 |  |  |  |  |  |  | 151 | 252 |
| Radakovic et al. (2021) (47) | 68 |  | 16 |  |  |  | 19 |  |  |  |  | 19 |  | 48 |  |  |  |  |  |  |  | 110 |
| Rastan et al. (2010) (48) | 300 |  |  |  |  |  | 90 | 19 | 28 |  |  | 28 |  | 336 |  | 111 |  |  |  |  | 189 | 389 |
| Ro et al. (2014) (49) |  |  |  |  |  |  |  |  |  |  |  |  |  | 26 |  |  |  |  |  |  | 135 | 180 |
| Roth et al. (2021) (50) | 166 |  |  |  |  |  |  |  | 40 |  | 117 |  |  | 210 |  |  |  |  |  |  |  | 200 |
| Rubino et al. (2018) (51) | 98 |  |  |  | 12 |  |  |  |  | 14 |  | 6 |  |  | 65 |  |  |  |  |  | 43 | 67 |
| Salna et al. (2021) (52) | 182 |  |  |  |  |  |  |  |  | 46 |  | 50 |  | 158 |  |  |  |  |  |  | 150 | 224 |
| Son et al. (2021) (53) |  |  | 24 |  |  |  |  |  |  |  |  | 21 | 2 |  |  |  |  |  |  |  | 52 | 68 |
| Toivonen et al. (2021) (54) |  |  |  |  |  |  | 149 | 47 | 84 | 18 |  |  | 12 | 409 |  | 285 |  | 179 |  |  |  | 502 |
| Vigneshwar et al. (2020) (55) | 194 |  |  |  |  |  |  | 37 | 46 |  |  | 87 |  | 385 |  |  |  |  |  |  |  | 457 |
| Wood et al. (2020) (56) | 121 | 15 |  | 25 | 17 | 11 |  | 7 | 9 |  | 37 | 20 |  | 54 |  | 16 | 28 | 13 |  |  | 101 | 133 |
| Wu et al. (2010) (57) |  |  |  |  |  |  |  |  |  |  |  | 11 |  | 46 |  |  |  |  |  |  | 43 | 64 |
| Yau et al. (2018) (58) |  |  |  |  |  |  |  |  |  |  |  | 34 | 3 |  |  |  |  |  |  |  |  | 92 |
| Zhigalov et al. (2019) (59) | 181 |  | 18 | 4 |  |  |  | 14 | 48 |  |  | 17 |  |  | 190 |  | 114 | 85 |  |  | 247 | 342 |
| ECMO: Extracorporeal membrane oxygenation, MODS: multiple organ dysfunction syndrome, CNS: central nervous system, | | | | | | | | | | | | | | | | | | | | | | |

# Table S7. Patient anticoagulation and ECMO characteristics of the included studies (n = 32)

| **Author** | **Anticoagulation (type)** | **ACT goal (s)** | **aPTT goal (s)** | **Pump type** | **UFH coating** |
| --- | --- | --- | --- | --- | --- |
| Aso et al. (2016) (28) | - | - | - | - | - |
| Aubin et al. (2017) (29) | - | - | - | - | - |
| Bonacchi et al. (2020) (30) | UFH | - | 40-50 | - | Yes |
| Cakici et al. (2017) (31) | - | - | - | Centrifugal | - |
| Choi et al. (2020) (32) | UFH | 150-180 | - | - | - |
| Elsharkawy et al. (2010) (33) | - | - | - | - | - |
| Fux et al. (2018) (34) | - | - | - | - | - |
| Karatolios et al. (2020) (35) | - | - | - | - | - |
| Laimoud et al. (2020) (36) | UFH | - | - | - | - |
| Lan et al. (2010) (37) | UFH | - | - | - | Yes |
| Li et al. (2015) (38) | UFH | 160-180 | - | Centrifugal | - |
| Liao et al. (2020) (39) | - | 180-200 | - | Centrifugal | Yes |
| Liem et al. (2020) (40) | - | - | - | - | - |
| Loforte et al. (2014) (41) | UFH | 140-160 | 50-70 | Centrifugal | Yes |
| Lunz et al. (2019) (42) | UFH | - | 55-65 | - | - |
| Masha et al. (2019) (43) | - | - | - | - | - |
| Mazzeffi et al. (2019) (44) | UFH | 180-200 | 60-80 | Centrifugal | - |
| McCloskey et al. (2021) (45) | UFH | - | 60-80 | - | - |
| Papadopoulos et al. (2015) (46) | UFH | - | 50-60 | Centrifugal | Yes |
| Radakovic et al. (2021) (47) | UFH | 160-180 | 50-70 | Centrifugal | - |
| Rastan et al. (2010) (48) | UFH | 160 | - | Centrifugal | Yes |
| Ro et al. (2014) (49) | UFH | 180-200 | - | Centrifugal | Yes |
| Roth et al. (2021) (50) | UFH | - | - | - | - |
| Rubino et al. (2018) (51) | - | - | - | - | - |
| Salna et al. (2021) (52) | UFH | - | 60-80 | - | - |
| Son et al. (2021) (53) | - | - | - | Centrifugal | Yes |
| Toivonen et al. (2021) (54) | - | - | - | - | - |
| Vigneshwar et al. (2020) (55) | - | - | - | - | - |
| Wood et al. (2020) (56) | UFH | 180-220 | 54-71 | Centrifugal | Yes |
| Wu et al. (2010) (57) | UFH | 180-200 | - | - | - |
| Yau et al. (2018) (58) | - | - | - | - | - |
| Zhigalov et al. (2019) (59) | - | - | - | - | - |

ACT, activated clotting time, aPTT, activated partial thromboplastin time, ECMO, extracorporeal membrane oxygenation, UFH, unfractionated heparin

# **Figure S1.** Influence analysis of studies reporting on in-hospital mortality. Baujat plot showing each study contribution to the overall heterogeneity. Aso et al. 2016 in the upper corner of the plot presents the study with the greatest impact on the explored effect and heterogeneity. Graphical display of study heterogeneity (GOSH) plot with potential outlier colored in cyan. The outlier is located in the upper part of effect size of other studies.


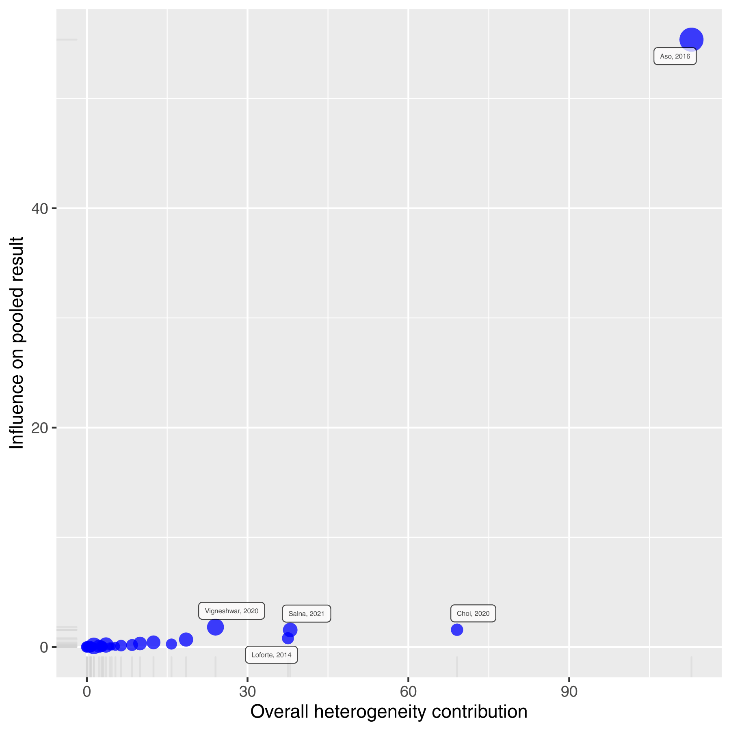

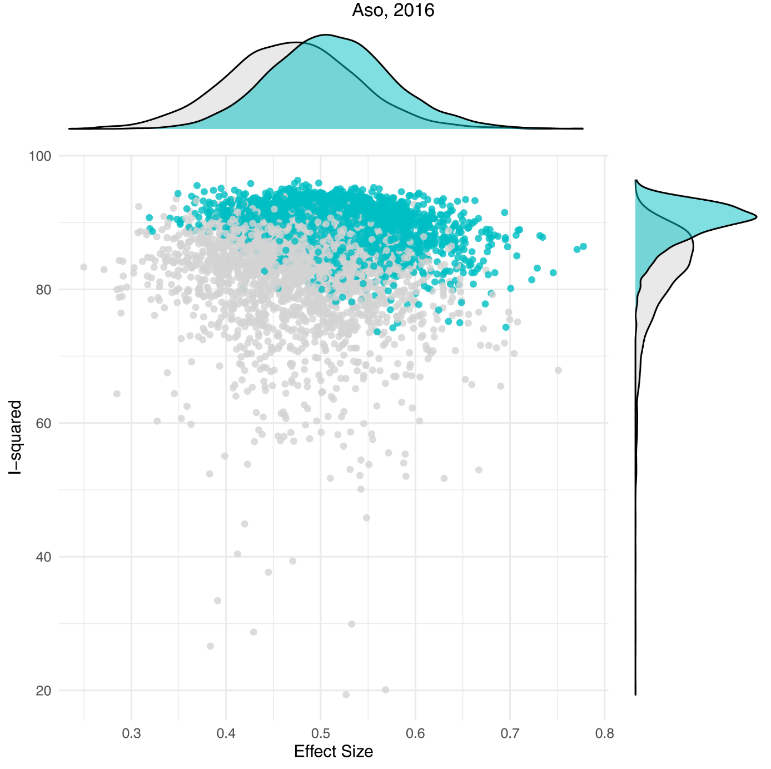


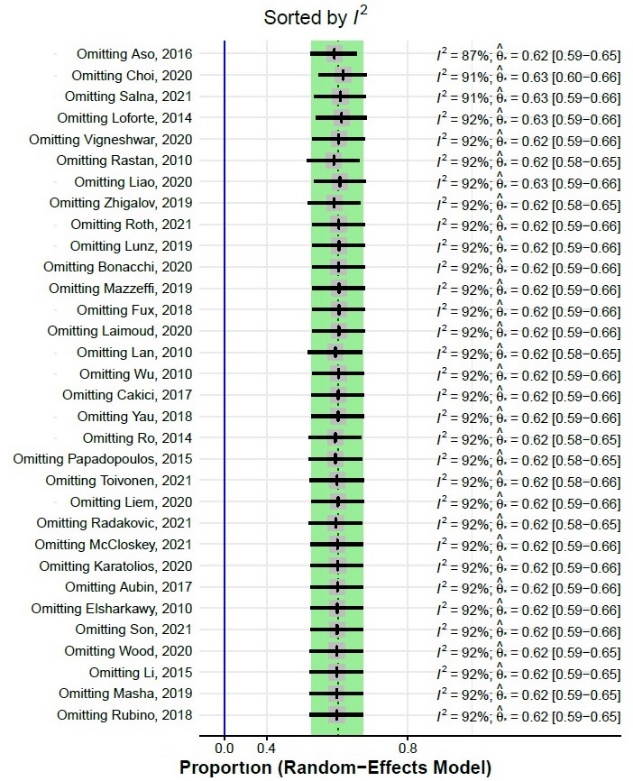

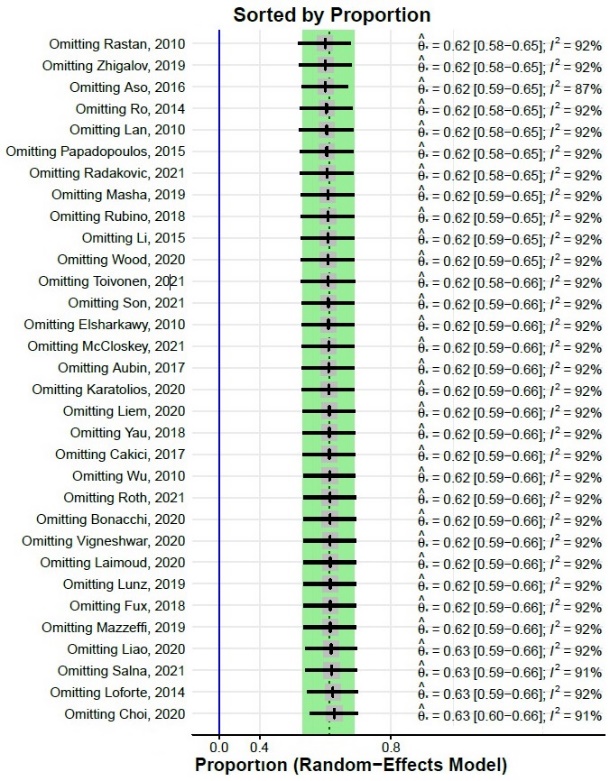


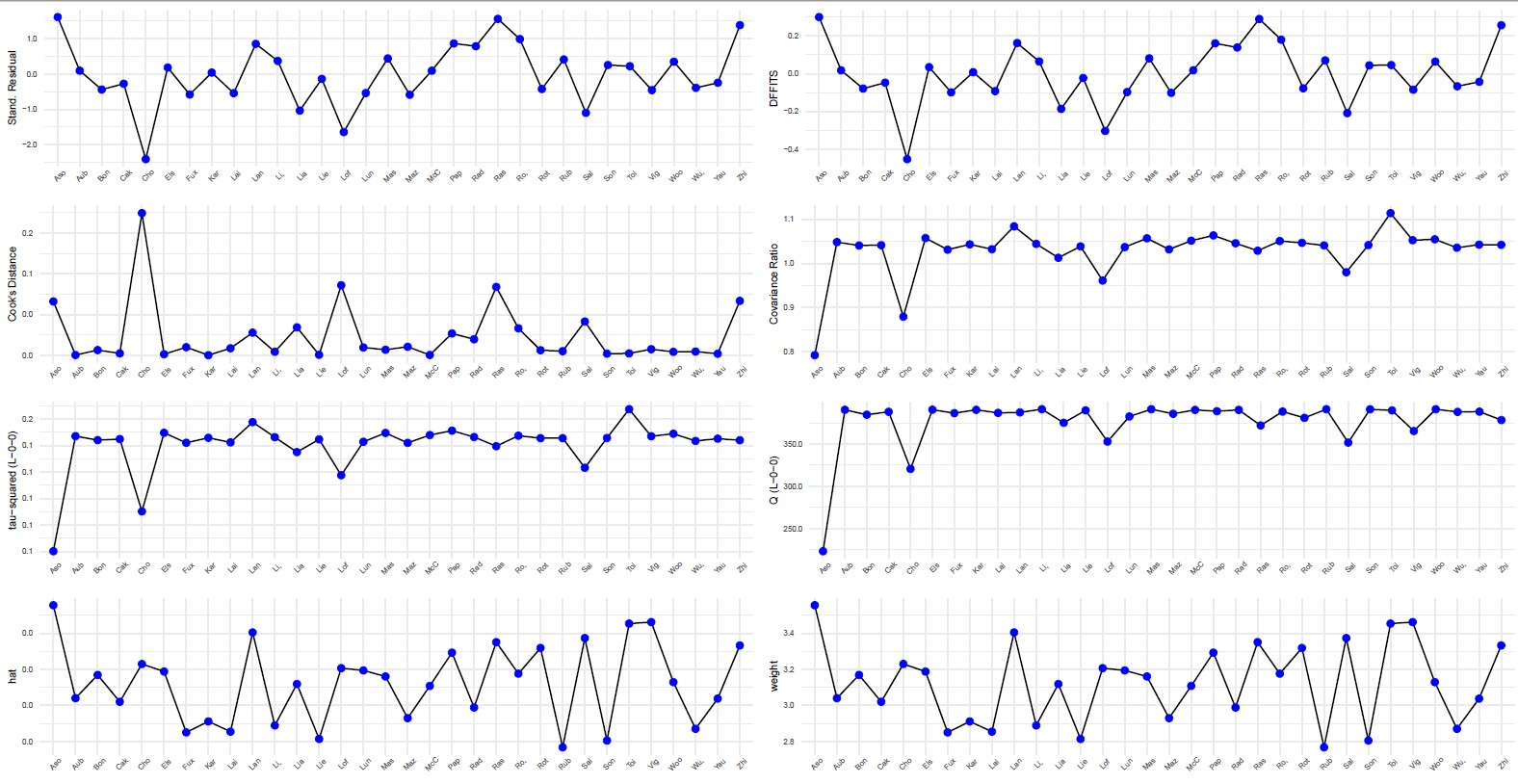


# Table S8. Influence analysis of studies reporting on in-hospital ECMO mortality

| **Analysis** | **Proportion (95% CI)** | **95% Prediction Interval** | ***I^2^* (95% CI)** |
| --- | --- | --- | --- |
| Main Analysis | 0.62 (0.59; 0.66) | 0.42; 0.79 | 92% (90; 94) |
| Influential case removed  (Aso et al., 2016) | 0.62 (0.59; 0.65) | 0.45; 0.76 | 87% (82; 90) |

# Table S9. Univariable meta-regression analyses

| **Outcome** | | **Data points** | **B-coefficient** | **Lower and upper 95% CI** | **P value** |
| --- | --- | --- | --- | --- | --- |
| Study characteristics | |  |  |  |  |
|  | Data collection (prospective or retrospective) | 32 | -0.188 | -0.497; 0.121 | 0.232 |
|  | Multicenter study setting | 32 | -0.058 | -0.425; 0.309 | 0.756 |
|  | Duration of recruitment period | 32 | 0.018 | -0.297; 0.333 | 0.910 |
|  | Recruitment period (threshold 10 years) | 32 | -0.183 | -0.522; 0.157 | 0.291 |
|  | Number of patients (threshold 200 patients) | 32 | 0.088 | -0.197; 0.373 | 0.545 |
|  | Inclusion of ECPR patients | 32 | 0.020 | -0.280; 0.319 | 0.128 |
| Patient demographic | |  |  |  |  |
|  | Gender | 32 | -0.003 | -0.022; 0.016 | 0.736 |
|  | Age (mean in years) | 23 | 0.018 | -0.007; 0.043 | 0.152 |
|  | Age (threshold 60 years) | 23 | 0.333 | 0.071; 0.596 | 0.013 |
|  | Body mass index(mean) | 10 | 0.025 | -0.115; 0.165 | 0.724 |
|  | ECMO support duration (mean, days) | 22 | -0.066 | -0.132; -0.001 | 0.048 |
|  | Cardiac arrest (ECPR) | 17 | -0.002 | -0.013; 0.010 | 0.795 |
| Bleeding | |  |  |  |  |
|  | Any bleeding | 15 | 0.007 | -0.005; 0.019 | 0.264 |
|  | Major bleeding | 13 | 0.005 | -0.013; 0.022 | 0.585 |
|  | Cannulation site bleeding | 8 | -0.030 | -0.047; -0.013 | 0.001 |
|  | Gastrointestinal bleeding | 6 | -0.035 | -0.081; 0.012 | 0.141 |
|  | Cardiac tamponade | 3 | -0.028 | -0.130; 0.073 | 0.586 |
| Stroke | |  |  |  |  |
|  | CNS complications (not specified) | 7 | 0.041 | -0.045; 0.127 | 0.352 |
|  | Hemorrhagic stroke | 12 | -0.022 | -0.051; 0.006 | 0.120 |
|  | Ischemic stroke | 13 | -0.011 | -0.039; 0.016 | 0.426 |
|  | Stroke (not defined as bleeding or ischemia) | 10 | -0.014 | -0.039; 0.012 | 0.301 |
| Thrombosis | |  |  |  |  |
|  | Any thrombosis | 5 | -0.001 | -0.014; 0.014 | 0.955 |
|  | Limb ischemia | 23 | 0.005 | -0.012; 0.021 | 0.598 |
|  | Limb amputation | 5 | 0.206 | -0.022; 0.435 | 0.077 |
| Renal failure | |  |  |  |  |
|  | Renal replacement therapy | 23 | -0.001 | -0.013; 0.011 | 0.849 |
|  | Renal failure | 7 | 0.001 | -0.011; 0.012 | 0.926 |
| Infections | |  |  |  |  |
|  | Infection (not specified) | 4 | 0.033 | 0.006; 0.060 | 0.017 |
|  | Pneumonia | 8 | -0.005 | -0.020; 0.010 | 0.486 |
|  | Sepsis | 9 | -0.011 | -0.048; 0.027 | 0.575 |
|  | MODS | 3 | -0.021 | -0.062; 0.020 | 0.322 |

ECPR, extracorporeal cardiopulmonary reanimation; ECMO, extracorporeal membrane oxygenation; CNS, central nervous system; MODS, multiple organ dysfunction syndrome.

# Figure S2. Meta-regression: scattered-plot of the relationship between age and in-hospital mortality


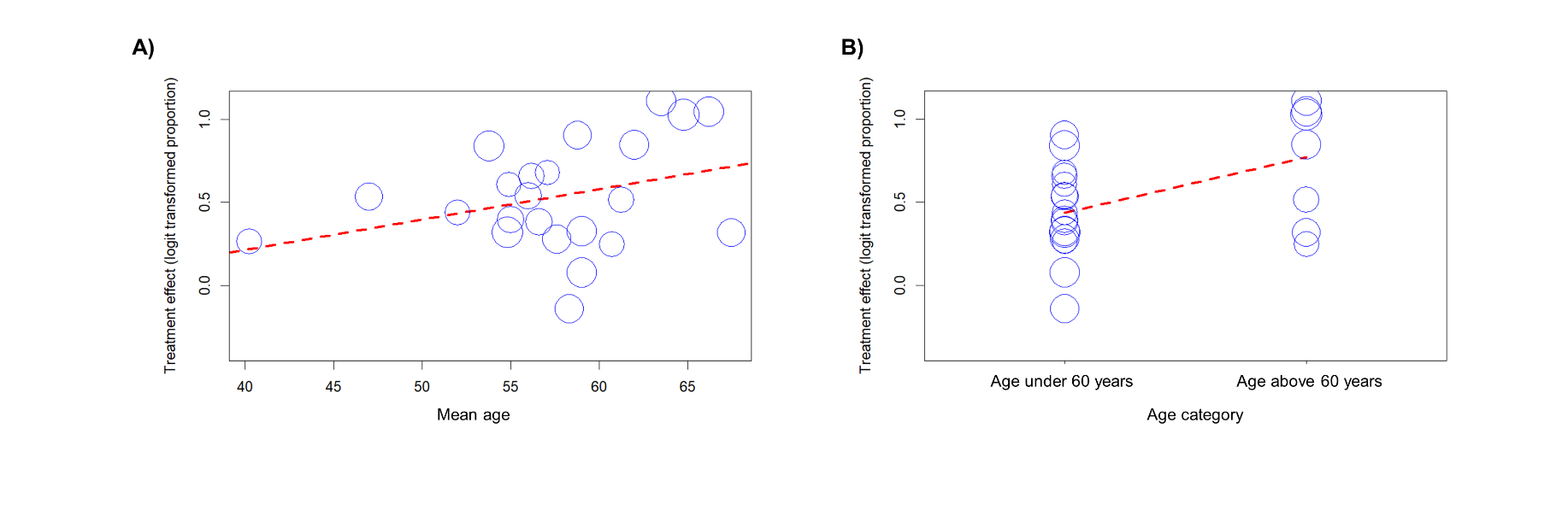


**A)** Details of meta-regression model results (mean age):

|  | **Coefficients** | **Standard error** | **P value** | **Lower bound 95% CI** | **Upper bound 95% CI** |
| --- | --- | --- | --- | --- | --- |
| **Intercept** | -0.518 | 0.741 | 0.485 | -1.97 | 0.93 |
| **Mean age** | 0.018 | 0.013 | 0.152 | -0.01 | 0.04 |

**B)** Details of meta-regression model results (categorical variable: age over 60 years):

|  | **Coefficients** | **Standard error** | **P value** | **Lower bound 95% CI** | **Upper bound 95% CI** |
| --- | --- | --- | --- | --- | --- |
| **Intercept** | 0.436 | 0.076 | <0.001 | 0.29 | 0.59 |
| **Age over 60 years** | 0.333 | 0.134 | 0.013 | 0.07 | 0.60 |

# References

1. Chen SW, Tsai FC, Lin YS, Chang CH, Chen DY, Chou AH, et al. Long-term outcomes of extracorporeal membrane oxygenation support for postcardiotomy shock. The Journal of thoracic and cardiovascular surgery. 2017;154(2):469-77.e2.

2. Chen CY, Tsai J, Hsu TY, Lai WY, Chen WK, Muo CH, et al. ECMO Used in a Refractory Ventricular Tachycardia and Ventricular Fibrillation Patient: A National Case-Control Study. Medicine. 2016;95(13):e3204.

3. Chen SW, Lu YA, Lee CC, Chou AH, Wu VC, Chang SW, et al. Long-term outcomes after extracorporeal membrane oxygenation in patients with dialysis-requiring acute kidney injury: A cohort study. PloS one. 2019;14(3):e0212352.

4. Chung M, Cabezas FR, Nunez JI, Kennedy KF, Rick K, Rycus P, et al. Hemocompatibility-Related Adverse Events and Survival on Venoarterial Extracorporeal Life Support: An ELSO Registry Analysis. JACC Heart failure. 2020;8(11):892-902.

5. Coutance G, Jacob N, Demondion P, Nguyen LS, Bouglé A, Bréchot N, et al. Favorable Outcomes of a Direct Heart Transplantation Strategy in Selected Patients on Extracorporeal Membrane Oxygenation Support. Critical care medicine. 2020;48(4):498-506.

6. Dangers L, Bréchot N, Schmidt M, Lebreton G, Hékimian G, Nieszkowska A, et al. Extracorporeal Membrane Oxygenation for Acute Decompensated Heart Failure. Critical care medicine. 2017;45(8):1359-66.

7. Distelmaier K, Wiedemann D, Lampichler K, Toth D, Galli L, Haberl T, et al. Interdependence of VA-ECMO output, pulmonary congestion and outcome after cardiac surgery. European journal of internal medicine. 2020;81:67-70.

8. Gass A, Palaniswamy C, Aronow WS, Kolte D, Khera S, Ahmad H, et al. Peripheral venoarterial extracorporeal membrane oxygenation in combination with intra-aortic balloon counterpulsation in patients with cardiovascular compromise. Cardiology. 2014;129(3):137-43.

9. Grandin EW, Nunez JI, Willar B, Kennedy K, Rycus P, Tonna JE, et al. Mechanical Left Ventricular Unloading in Patients Undergoing Venoarterial Extracorporeal Membrane Oxygenation. J Am Coll Cardiol. 2022;79(13):1239-50.

10. Hayanga AJ, Aboagye J, Esper S, Shigemura N, Bermudez CA, D'Cunha J, et al. Extracorporeal membrane oxygenation as a bridge to lung transplantation in the United States: an evolving strategy in the management of rapidly advancing pulmonary disease. The Journal of thoracic and cardiovascular surgery. 2015;149(1):291-6.

11. Huckaby LV, Hickey G, Sultan I, Kilic A. Improvements in Functional Status Among Survivors of Orthotopic Heart Transplantation Following High-risk Bridging Modalities. Transplantation. 2021;105(9):2097-103.

12. Kashiura M, Yasuda H, Kishihara Y, Tominaga K, Nishihara M, Hiasa K-i, et al. Association between short-term neurological outcomes and extreme hyperoxia in patients with out-of-hospital cardiac arrest who underwent extracorporeal cardiopulmonary resuscitation: a retrospective observational study from a multicenter registry. BMC Cardiovascular Disorders. 2022;22(1):163.

13. Kowalewski M, Zieliński K, Brodie D, MacLaren G, Whitman G, Raffa GM, et al. Venoarterial Extracorporeal Membrane Oxygenation for Postcardiotomy Shock-Analysis of the Extracorporeal Life Support Organization Registry. Critical care medicine. 2021;49(7):1107-17.

14. Lee D-S, Chung CR, Jeon K, Park C-M, Suh GY, Song YB, et al. Survival After Extracorporeal Cardiopulmonary Resuscitation on Weekends in Comparison With Weekdays. The Annals of thoracic surgery. 2016;101(1):133-40.

15. Loforte A, Pilato E, Martin Suarez S, Folesani G, Jafrancesco G, Castrovinci S, et al. [Extracorporeal membrane oxygenation for the treatment of refractory cardiogenic shock in adults: strategies, results, and predictors of mortality]. Giornale italiano di cardiologia (2006). 2014;15(10):577-85.

16. Loyaga-Rendon RY, Boeve T, Tallaj J, Lee S, Leacche M, Lotun K, et al. Extracorporeal Membrane Oxygenation as a Bridge to Durable Mechanical Circulatory Support: An Analysis of the STS-INTERMACS Database. Circulation Heart failure. 2020;13(3):e006387.

17. Mariscalco G, Fiore A, Ragnarsson S, El-Dean Z, Jónsson K, Dalén M, et al. Venoarterial Extracorporeal Membrane Oxygenation After Surgical Repair of Type A Aortic Dissection. The American journal of cardiology. 2020;125(12):1901-5.

18. Mastoris I, Tonna JE, Hu J, Sauer AJ, Haglund NA, Rycus P, et al. Use of Extracorporeal Membrane Oxygenation as Bridge to Replacement Therapies in Cardiogenic Shock: Insights From the Extracorporeal Life Support Organization. Circulation Heart failure. 2022;15(1):e008777.

19. Seong SW, Jin G, Kim M, Ahn KT, Yang JH, Gwon HC, et al. Comparison of in-hospital outcomes of patients with vs. without ischaemic cardiomyopathy undergoing veno-arterial-extracorporeal membrane oxygenation. ESC heart failure. 2021;8(4):3308-15.

20. Thiagarajan RR, Brogan TV, Scheurer MA, Laussen PC, Rycus PT, Bratton SL. Extracorporeal membrane oxygenation to support cardiopulmonary resuscitation in adults. The Annals of thoracic surgery. 2009;87(3):778-85.

21. Tsai TY, Chien H, Tsai FC, Pan HC, Yang HY, Lee SY, et al. Comparison of RIFLE, AKIN, and KDIGO classifications for assessing prognosis of patients on extracorporeal membrane oxygenation. Journal of the Formosan Medical Association = Taiwan yi zhi. 2017;116(11):844-51.

22. Vallabhajosyula S, Prasad A, Bell MR, Sandhu GS, Eleid MF, Dunlay SM, et al. Extracorporeal Membrane Oxygenation Use in Acute Myocardial Infarction in the United States, 2000 to 2014. Circulation Heart failure. 2019;12(12):e005929.

23. Wang L, Yang F, Zhang S, Li C, Du Z, Rycus P, et al. Percutaneous versus surgical cannulation for femoro-femoral VA-ECMO in patients with cardiogenic shock: Results from the Extracorporeal Life Support Organization Registry. The Journal of Heart and Lung Transplantation. 2022;41(4):470-81.

24. Yeo HJ, Yoon SH, Jeon D, Kim YS, Cho WH, Kim D, et al. The Utility of Preemptive Distal Perfusion Cannulation During Peripheral Venoarterial Extracorporeal Membrane Oxygenation Support. Journal of interventional cardiology. 2016;29(4):431-6.

25. Yen CC, Kao CH, Tsai CS, Tsai SH. Identifying the Risk Factor and Prevention of Limb Ischemia in Extracorporeal Membrane Oxygenation with Femoral Artery Cannulation. The heart surgery forum. 2018;21(1):E018-e22.

26. Yu K, Long C, Hei F, Li J, Liu J, Ji B, et al. Clinical evaluation of two different extracorporeal membrane oxygenation systems: a single center report. Artificial organs. 2011;35(7):733-7.

27. Yusuff H, Biancari F, Jónsson K, Ragnarsson S, Dalén M, Fux T, et al. Outcome of Repeat Venoarterial Extracorporeal Membrane Oxygenation in Postcardiotomy Cardiogenic Shock. Journal of cardiothoracic and vascular anesthesia. 2021;35(12):3620-5.

28. Aso S, Matsui H, Fushimi K, Yasunaga H. In-hospital mortality and successful weaning from venoarterial extracorporeal membrane oxygenation: analysis of 5,263 patients using a national inpatient database in Japan. Critical care (London, England). 2016;20:80.

29. Aubin H, Petrov G, Dalyanoglu H, Richter M, Saeed D, Akhyari P, et al. Four-year experience of providing mobile extracorporeal life support to out-of-center patients within a suprainstitutional network-Outcome of 160 consecutively treated patients. Resuscitation. 2017;121:151-7.

30. Bonacchi M, Cabrucci F, Bugetti M, Dokollari A, Parise O, Sani G, et al. Outcomes' predictors in Post-Cardiac Surgery Extracorporeal Life Support. An observational prospective cohort study. International journal of surgery (London, England). 2020;82:56-63.

31. Cakici M, Ozcinar E, Baran C, Bermede AO, Sarıcaoglu MC, Inan MB, et al. A retrospective cohort analysis of percutaneous versus side-graft perfusion techniques for veno-arterial extracorporeal membrane oxygenation in patients with refractory cardiogenic shock. Perfusion. 2017;32(5):363-71.

32. Choi KH, Yang JH, Hong D, Park TK, Lee JM, Song YB, et al. Optimal Timing of Venoarterial-Extracorporeal Membrane Oxygenation in Acute Myocardial Infarction Patients Suffering From Refractory Cardiogenic Shock. Circulation journal : official journal of the Japanese Circulation Society. 2020;84(9):1502-10.

33. Elsharkawy HA, Li L, Esa WA, Sessler DI, Bashour CA. Outcome in patients who require venoarterial extracorporeal membrane oxygenation support after cardiac surgery. Journal of cardiothoracic and vascular anesthesia. 2010;24(6):946-51.

34. Fux T, Holm M, Corbascio M, Lund LH, van der Linden J. Venoarterial extracorporeal membrane oxygenation for postcardiotomy shock: Risk factors for mortality. The Journal of thoracic and cardiovascular surgery. 2018;156(5):1894-902.e3.

35. Karatolios K, Chatzis G, Markus B, Luesebrink U, Ahrens H, Divchev D, et al. Comparison of mechanical circulatory support with venoarterial extracorporeal membrane oxygenation or Impella for patients with cardiogenic shock: a propensity-matched analysis. Clinical research in cardiology : official journal of the German Cardiac Society. 2021;110(9):1404-11.

36. Laimoud M, Alanazi M. The clinical significance of blood lactate levels in evaluation of adult patients with veno-arterial extracorporeal membrane oxygenation. The Egyptian heart journal : (EHJ) : official bulletin of the Egyptian Society of Cardiology. 2020;72(1):74.

37. Lan C, Tsai PR, Chen YS, Ko WJ. Prognostic factors for adult patients receiving extracorporeal membrane oxygenation as mechanical circulatory support--a 14-year experience at a medical center. Artificial organs. 2010;34(2):E59-64.

38. Li CL, Wang H, Jia M, Ma N, Meng X, Hou XT. The early dynamic behavior of lactate is linked to mortality in postcardiotomy patients with extracorporeal membrane oxygenation support: A retrospective observational study. The Journal of thoracic and cardiovascular surgery. 2015;149(5):1445-50.

39. Liao X, Cheng Z, Wang L, Li B, Huang W, Wen J, et al. Vascular Complications of Lower Limb Ischemia in Patients with Femoral Venoarterial Extracorporeal Membrane Oxygenation. The heart surgery forum. 2020;23(3):E305-e9.

40. Liem S, Cavarocchi NC, Hirose H. Comparing in-patient extracorporeal cardiopulmonary resuscitation to standard cardiac treatment group of extracorporeal membrane oxygenation patients: 8 years of experience at a single institution. Perfusion. 2020;35(1):73-81.

41. Loforte A, Marinelli G, Musumeci F, Folesani G, Pilato E, Martin Suarez S, et al. Extracorporeal membrane oxygenation support in refractory cardiogenic shock: treatment strategies and analysis of risk factors. Artificial organs. 2014;38(7):E129-41.

42. Lunz D, Philipp A, Müller T, Pfister K, Foltan M, Rupprecht L, et al. Ischemia-related vascular complications of percutaneously initiated venoarterial extracorporeal membrane oxygenation: Indication setting, risk factors, manifestation and outcome. Journal of critical care. 2019;52:58-62.

43. Masha L, Peerbhai S, Boone D, Shobayo F, Ghotra A, Akkanti B, et al. Yellow Means Caution: Correlations Between Liver Injury and Mortality with the Use of VA-ECMO. ASAIO journal (American Society for Artificial Internal Organs : 1992). 2019;65(8):812-8.

44. Mazzeffi MA, Tanaka K, Roberts A, Rector R, Menaker J, Kon Z, et al. Bleeding, Thrombosis, and Transfusion With Two Heparin Anticoagulation Protocols in Venoarterial ECMO Patients. Journal of cardiothoracic and vascular anesthesia. 2019;33(5):1216-20.

45. McCloskey CG, Engoren MC. Transfusion and its association with mortality in patients receiving veno-arterial extracorporeal membrane oxygenation. Journal of critical care. 2022;68:42-7.

46. Papadopoulos N, Marinos S, El-Sayed Ahmad A, Keller H, Meybohm P, Zacharowski K, et al. Risk factors associated with adverse outcome following extracorporeal life support: analysis from 360 consecutive patients. Perfusion. 2015;30(4):284-90.

47. Radakovic D, Hamouda K, Penov K, Bening C, Sayed S, Gietzen C, et al. Central Versus Peripheral Arterial Cannulation for Veno-Arterial Extracorporeal Membrane Oxygenation in Post-Cardiotomy Patients. ASAIO journal (American Society for Artificial Internal Organs : 1992). 2021;67(1):67-73.

48. Rastan AJ, Dege A, Mohr M, Doll N, Falk V, Walther T, et al. Early and late outcomes of 517 consecutive adult patients treated with extracorporeal membrane oxygenation for refractory postcardiotomy cardiogenic shock. The Journal of thoracic and cardiovascular surgery. 2010;139(2):302-11, 11.e1.

49. Ro SK, Kim JB, Jung SH, Choo SJ, Chung CH, Lee JW. Extracorporeal life support for cardiogenic shock: influence of concomitant intra-aortic balloon counterpulsation. European journal of cardio-thoracic surgery : official journal of the European Association for Cardio-thoracic Surgery. 2014;46(2):186-92; discussion 92.

50. Roth S, Jansen C, M'Pembele R, Stroda A, Boeken U, Akhyari P, et al. Fibrinogen-Albumin-Ratio is an independent predictor of thromboembolic complications in patients undergoing VA-ECMO. Scientific reports. 2021;11(1):16648.

51. Rubino A, Costanzo D, Stanszus D, Valchanov K, Jenkins D, Sertic F, et al. Central Veno-Arterial Extracorporeal Membrane Oxygenation (C-VA-ECMO) After Cardiothoracic Surgery: A Single-Center Experience. Journal of cardiothoracic and vascular anesthesia. 2018;32(3):1169-74.

52. Salna M, Fried J, Kaku Y, Brodie D, Sayer G, Uriel N, et al. Obesity is not a contraindication to veno-arterial extracorporeal life support. European journal of cardio-thoracic surgery : official journal of the European Association for Cardio-thoracic Surgery. 2021;60(4):831-8.

53. Son AY, Khanh LN, Joung HS, Guerra A, Karim AS, McGregor R, et al. Limb ischemia and bleeding in patients requiring venoarterial extracorporeal membrane oxygenation. Journal of vascular surgery. 2021;73(2):593-600.

54. Toivonen F, Biancari F, Dalén M, Dell'Aquila AM, Jónsson K, Fiore A, et al. Neurologic Injury in Patients Treated With Extracorporeal Membrane Oxygenation for Postcardiotomy Cardiogenic Shock. Journal of cardiothoracic and vascular anesthesia. 2021;35(9):2669-80.

55. Vigneshwar NG, Kohtz PD, Lucas MT, Bronsert M, M JW, M FM, et al. Clinical predictors of in-hospital mortality in venoarterial extracorporeal membrane oxygenation. Journal of cardiac surgery. 2020;35(10):2512-21.

56. Wood KL, Ayers B, Gosev I, Kumar N, Melvin AL, Barrus B, et al. Venoarterial-Extracorporeal Membrane Oxygenation Without Routine Systemic Anticoagulation Decreases Adverse Events. The Annals of thoracic surgery. 2020;109(5):1458-66.

57. Wu MY, Lin PJ, Lee MY, Tsai FC, Chu JJ, Chang YS, et al. Using extracorporeal life support to resuscitate adult postcardiotomy cardiogenic shock: treatment strategies and predictors of short-term and midterm survival. Resuscitation. 2010;81(9):1111-6.

58. Yau P, Xia Y, Shariff S, Jakobleff WA, Forest S, Lipsitz EC, et al. Factors Associated with Ipsilateral Limb Ischemia in Patients Undergoing Femoral Cannulation Extracorporeal Membrane Oxygenation. Annals of vascular surgery. 2019;54:60-5.

59. Zhigalov K, Sá MPBO, Safonov D, Zagitov I, Alofesh A, Pavlova V, et al. Clinical outcomes of venoarterial extracorporeal life support in 462 patients: Single-center experience. 2020;44(6):620-7.
